# The NRF2-mediated oxidative stress response pathway is associated with tumor cell resistance to arsenic trioxide across the NCI-60 panel

**DOI:** 10.1186/1755-8794-3-37

**Published:** 2010-08-13

**Authors:** Qian Liu, Hao Zhang, Lisa Smeester, Fei Zou, Matt Kesic, Ilona Jaspers, Jingbo Pi, Rebecca C Fry

**Affiliations:** 1 Department of Environmental Sciences and Engineering, Gillings School of Global Public Health, University of North Carolina, Chapel Hill, NC, USA; 2 Department of Biostatistics, Gillings School of Global Public Health, University of North Carolina, Chapel Hill, NC, USA; 3 Center for Environmental Medicine, Asthma, and Lung Biology, School of Medicine, University of North Carolina, Chapel Hill, NC, USA; 4 The Hamner Institutes for Health Sciences, Research Triangle Park, NC, USA; 5 Department of Environmental Health, School of Public Health, Fudan University, Shanghai 200032, P.R. China

## Abstract

**Background:**

Drinking water contaminated with inorganic arsenic is associated with increased risk for different types of cancer. Paradoxically, arsenic trioxide can also be used to induce remission in patients with acute promyelocytic leukemia (APL) with a success rate of approximately 80%. A comprehensive study examining the mechanisms and potential signaling pathways contributing to the anti-tumor properties of arsenic trioxide has not been carried out.

**Methods:**

Here we applied a systems biology approach to identify gene biomarkers that underlie tumor cell responses to arsenic-induced cytotoxicity. The baseline gene expression levels of 14,500 well characterized human genes were associated with the GI_50_ data of the NCI-60 tumor cell line panel from the developmental therapeutics program (DTP) database. Selected biomarkers were tested *in vitro* for the ability to influence tumor susceptibility to arsenic trioxide.

**Results:**

A significant association was found between the baseline expression levels of 209 human genes and the sensitivity of the tumor cell line panel upon exposure to arsenic trioxide. These genes were overlayed onto protein-protein network maps to identify transcriptional networks that modulate tumor cell responses to arsenic trioxide. The analysis revealed a significant enrichment for the oxidative stress response pathway mediated by nuclear factor erythroid 2-related factor 2 (NRF2) with high expression in arsenic resistant tumor cell lines. The role of the NRF2 pathway in protecting cells against arsenic-induced cell killing was validated in tumor cells using shRNA-mediated knock-down.

**Conclusions:**

In this study, we show that the expression level of genes in the NRF2 pathway serve as potential gene biomarkers of tumor cell responses to arsenic trioxide. Importantly, we demonstrate that tumor cells that are deficient for NRF2 display increased sensitivity to arsenic trioxide. The results of our study will be useful in understanding the mechanism of arsenic-induced cytotoxicity in cells, as well as the increased applicability of arsenic trioxide as a chemotherapeutic agent in cancer treatment.

## Background

Arsenic poisoning is a global health issue and epidemiological studies indicate that chronic arsenic exposure in drinking water is linked to increased risk for various types of cancer [1-3]. More than 40 million people are exposed to drinking water with arsenic levels that far exceed the guideline established by the World Health Organization (WHO) and the limit acceptable by the US Environmental Protection Agency (EPA) of 10 ppb [4,5].

In contrast to its carcinogenic properties, arsenic trioxide can also be used as a clinically active agent to induce complete remission of acute promyelocytic leukemia (APL). The first clinical trial on arsenic trioxide treatment of relapsed APL patients after resistance to all-trans-retinoic acid (ATRA) treatment was carried out in China with a complete remission rate of 72% [6]. In another NCI-sponsored cancer and leukemia study, 77% of newly diagnosed APL patients who received combined chemotherapy and single arsenic trioxide treatment remained in remission 3 years after diagnosis [7]. It is well accepted that arsenic trioxide results in apoptosis in multidrug resistant APL cells [8,9]. A primary mechanism associated with arsenic-trioxide's effectiveness in treating APL is related to the ability to degrade and cleave the promyelocytic leukemia retinoic acid receptor-α (PML-RARα) oncoprotein [10]. As well, arsenic-induced apoptosis has been linked to the generation of hydrogen peroxide [11] and Bcl-2 down-regulation [12]. However, a comprehensive study examining the mechanisms and potential signaling pathways contributing to its anti-tumor properties has not been carried out.

In this research, we set out to identify gene biomarkers that are highly correlated with tumor cell responses to arsenic-induced cytotoxicity. The rationale was based on studies demonstrating that gene biomarkers can be used as predictors of tumor cell responses to therapeutic treatments [13,14]. The NCI-60 cell panel contains 60 human tumor cell lines that originate from nine different tumor types. Based on our systems biology analysis of the NCI-60 cell panel, we identified 209 human genes whose baseline expression levels were statistically associated with tumor cell susceptibility to arsenic trioxide. By integrating the gene biomarkers with known protein-protein networks, we show that the NRF2-mediated oxidative stress response pathway is significantly associated with tumor cell resistance to arsenic-induced cytotoxicity. Importantly, by generating tumor cells deficient for the expression of NRF2, we validate our computational prediction and demonstrate that, indeed, this pathway is involved in tumor cell resistance to arsenic trioxide. Moreover, our results also indicate possible interactions between NRF2 and NFκB, which might contribute to the cellular resistance upon exposure to arsenic trioxide. Results from this study will help us to better understand the genes that influence the dual properties of arsenic trioxide as a human carcinogen and an effective chemotherapeutic agent.

## Methods

### *In vitro *arsenic trioxide screening data

The arsenic trioxide GI_50 _data were obtained from the Developmental Therapeutics Program (DTP) database at http://dtp.nci.nih.gov. The NCI-60 human tumor cell panel was used in the *in vitro *cell line screening project (IVCLSP) under the DTP program, where 59 cell lines in the NCI-60 cell panel were exposed to arsenic trioxide for 48 hours and growth inhibition of 50% (GI_50_) was recorded as the drug concentration resulting in a 50% reduction in the net protein increase in control cells during the drug incubation [15]. Cell lines were numbered from 1 to 59, corresponding to the increased cellular sensitivity to arsenic trioxide (Additional File 1).

### Baseline gene expression data

The baseline gene expression data was from a previous publication [16] and the data are available online at http://discover.nci.nih.gov/. Using 59 cell lines of the NCI-60 human tumor cell panel, the investigators measured the baseline gene expression levels of 22,238 gene probes (representative of 14,500 human genes) using the Affymetrix HG-U133A chip [17].

### Significance Analysis of Microarrays (SAM)

Significance analysis of microarrays (SAM) [18] was used to identify the association between the baseline gene expression levels and tumor cell responses (e.g. resistance or sensitivity) to arsenic trioxide. Specifically, SAM was used to identify statistically significant gene probes by carrying out gene specific t-tests and computing a score which measures the strength of the relationship between the expression of each gene (transcription profile) and the response variable (GI_50_). The use of permutation-based analysis accounts for correlations in genes and avoids parametric assumptions about the distribution of individual genes [19]. In this study, we set the false discovery rate (FDR) to 0.05 for declaring the significance of genes. According to data availability, 58 cell lines of the NCI-60 cell panel were included in our analysis - cell line 36 was excluded.

### Network analysis and pathway mapping

Molecular network analysis and pathway mapping were carried out using the Ingenuity Knowledge Base http://www.ingenuity.com, a repository database of molecular interactions, regulatory events, gene-to-phenotype associations, and chemical knowledge [20]. With this systems biology tool, we integrated differentially expressed genes with known molecular networks. Networks are algorithmically generated based on their connectivity. The functional analysis of a network identifies the biological functions and/or diseases that are most significantly enriched in the network using a Fisher's Exact test [21].

### *NRF2 *knock-down cell generation and real-time RT-PCR validation

The A549 lung carcinoma tumor cell line (cell line #2 in Additional File 1) was used to generate cells deficient for the expression of NRF2 using short hairpin RNAs (shRNAs). Additionally, a control shRNA that has a scrambled sequence with no genome targeting, but that controls for the activation of RNAi machinery was also infected into the tumor cell line. For the lentiviral-based shRNA transduction, MISSION shRNA lentiviral particles were obtained from Sigma. The lentiviral transduction of A549 cells with particles for shRNAs targeting *NRF2 *(SHVRS-NM_006164), scrambled non-target negative control (Scramble, SHC002V) or TurboGFP control (GFP, SHC003) was performed as described previously [22]. The cells were maintained in medium containing 3.0 μg/ml of puromycin. Knock-down of *NRF2 *was confirmed with real-time RT-PCR where expression was normalized to *18S*. Primer sequences used to amplify *NRF2 *(NM-006164), *NQO1 *(NM_000903), *β-ACTIN *(X00351) and housekeeping gene *18 S *(N87634) are as follows: (1) *NRF2*: forward: (ACCAGTGGATCTGCCAACTACTC) and reverse: (CTGCGCCAAAAGCTGCAT); (2) *NQO1*: forward (ACTGCCCTCTTGTGGTGCAT) and reverse: GCTCGGTCCAATCCCTTCAT; (3) *β-ACTIN: *forward (GTCCACCTTCCAGCAGATGTG); reverse (GCATTTGCGGTGGACGAT) and (4) *18S: *forward (CGCCCCAGCACTTTGG) and reverse (TTACCAGCGGATGGATGGA).

### Cytotoxicity assays

To measure arsenic-induced cytotoxicity in the knock-down cells relative to control cells, a non-Radioactive Cell-Proliferation Assay Kit was used (Promega, Madison, WI). A minimum of 5 replicates of 10,000 cells per well were plated in 96-well plates and allowed to adhere to the plate for 24 hrs, at which time the media was removed and replaced with fresh media containing arsenic trioxide. Cells were then incubated for an additional 24 hrs and cell viability was determined. Measurements are expressed as percent of untreated control (vehicle) of appropriate cells. As a second method to assess arsenic-induced cytotoxicity, the enzyme lactate dehydrogenase (LDH) was measured in control or exposed cells. Cells were exposed in biological duplicate to inorganic arsenic across a dose range for 24 hrs and cytotoxicity determined using LDH release. Measurements were acquired using a coupled enzymatic assay according to the supplier's instructions (Takara Bio Inc., Japan) and are represented as fold increase in LDH of *NRF2*-KD versus control.

## Results

### The baseline expression levels of 209 human genes are associated with tumor cell responses to arsenic trioxide

**Figure 1:**
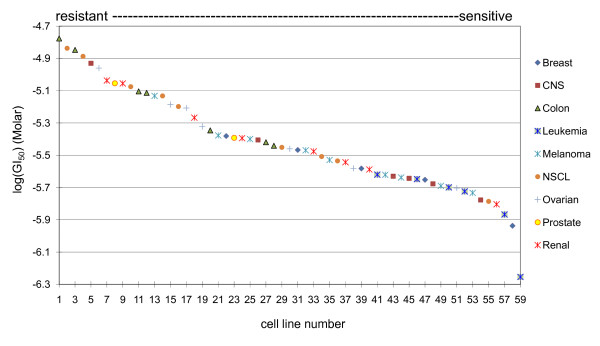
**A range of susceptibilities to arsenic trioxide across the NCI-60 tumor cell panel**. The GI_50 _data of 59 tumor cell lines screened for arsenic trioxide-induced cell death are displayed. A total of nine tumor types were screened, including: breast, central nervous system (CNS), colon, leukemia, melanoma, non-small cell lung (NSCL), ovarian, prostate, and renal tumors. For the complete list of tumor cell lines refer to Additional File 1.

We set out to identify gene biomarkers of tumor cell responses to arsenic trioxide. Using data obtained from the DTP database [23], it is clear that the NCI-60 human tumor cell lines show differential cytotoxicity responses upon exposure to arsenic trioxide (see Methods; Figure 1; Additional File 1). The baseline gene expression data for the tumor cell lines were derived from a previous study [16]. The baseline gene expression levels of more than 14,500 well characterized human genes were analyzed for the NCI-60 cell panel using the Affymetrix Human Genome Array U133A. Because of data availability, our study included 58 of the 60 human tumor cell lines.

**Figure 2:**
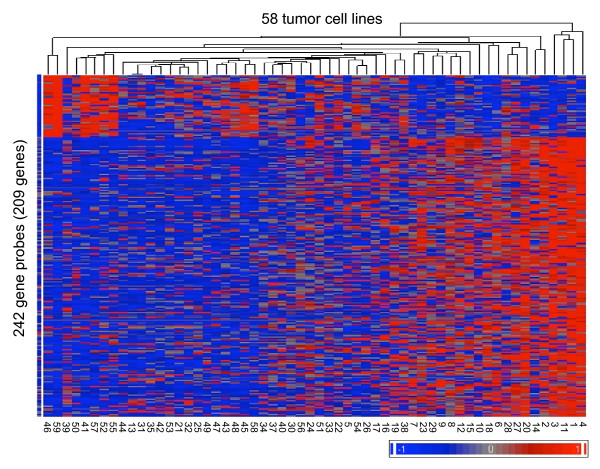
**Potential gene biomarkers of tumor cell susceptibility to arsenic trioxide**. A total of 209 genes (242 gene probes) were identified with significant expression association with tumor cell susceptibility to arsenic trioxide across 58 tumor cell lines (FDR < 0.05). Cell line numbers are displayed on the X-axis. For the complete list of tumor cell lines refer to Additional File 1. Gene expression values were mean centered and high relative expression is indicated in red and low relative expression indicated in blue.

To identify genes with expression levels associated with tumor cell susceptibility to arsenic trioxide, we applied a significance analysis of microarray (SAM) analysis [18] (see Methods). This resulted in the identification of 209 human genes (represented by 242 gene probes) whose baseline expression levels were statistically associated with tumor cell responses to arsenic trioxide (FDR < 0.05) (Figure 2; Additional File 2). Of the 209 genes, 169 genes had high expression in arsenic resistant tumor cell lines, whereas the other 40 genes had high expression in arsenic sensitive tumor cell lines.

### Arsenic susceptibility genes are enriched for numerous biological processes including tumorigenesis

To identify biological processes associated with tumor cell responses to arsenic trioxide, we analyzed the 209 arsenic susceptibility-associated genes for network interactions (see Methods). A total of 188 of the 209 genes were eligible (e.g. present in the database) for network generation.

**Figure 3:**
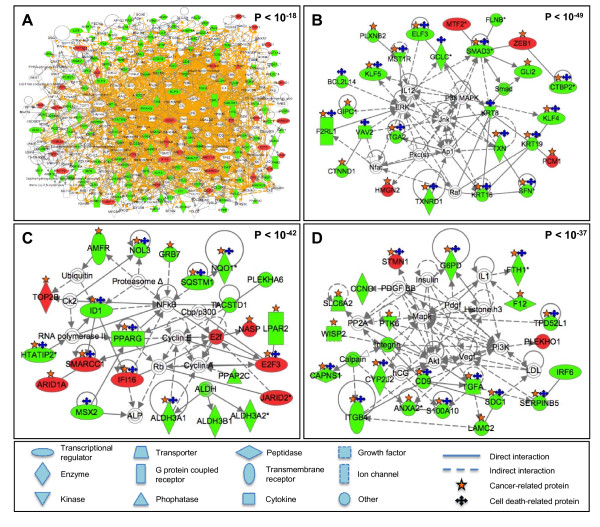
**Molecular interactomes and sub-networks associated with tumor cell susceptibility to arsenic trioxide**. (A) A large arsenic-susceptibility interactome containing 317 proteins was identified. (B-D) The three most significant cancer and cell death enriched sub-networks within the large interactome were identified. Networks are displayed with symbols representing encoded proteins corresponding to their RNA transcripts that were either highly expressed in arsenic resistant cell lines (green symbols), highly expressed in arsenic sensitive cell lines (red symbols), or associated to the modified transcripts (white symbols). P-values representing the statistical significance of networks are shown.

Through network mapping, we identified a large interactome associated with cellular response to arsenic trioxide (p < 10^-18^), which contained a total 317 proteins (Figure 3A). This large interactome is enriched for biological processes related to tumorigenesis, including cancer, cell death, cellular movement, cell-to-cell signaling and interaction, cellular growth and proliferation, and tumor morphology (Additional File 3 and 4). Within this large arsenic-susceptibility-associated interactome, we identified 10 smaller, more focused sub-networks with p values < 10^-18^. The top three sub-networks range in significance from p < 10^-37 ^to p < 10^-49^, and they are enriched for 64 biological functions, among the most significant are cancer and cell death (Figure 3; Additional File 5).

The three sub-networks contain a total of 105 unique proteins. Of these, 70 were associated with tumor cell susceptibility to arsenic trioxide (Figure 3; Additional File 3). Within the 70 arsenic-susceptibility-associated proteins, 13 had high expression in arsenic-sensitive cell lines (19%) and 57 genes had high expression in arsenic-resistant cell lines (81%) (Additional File 3). Of these 70 proteins, 54 are associated with cancer, and 40 are associated with cell death (Figure 3).

The three sub-networks were found to be enriched for 18 transcriptional regulators (Table 1). Within these transcription factors, ID1 is known for its function in tumorigenesis [24] and also a possible therapeutic target for cancer treatment [25]. Other than these transcription factors, we also discovered protein complexes as integrated nodes in the three sub-networks that are associated with cellular response to arsenic-induced cytotoxicity, including activator protein 1 (Ap1) [26] and nuclear factor kappa B (NFκB) [27].

**Table 1:** Transcription factors identified within the sub-networks

Transcription Factor	Description	Sub-network
ELF3	E74-like factor 3	1

MTF2	metal response element binding transcription factor 2	1

ZEB1	zinc finger E-box binding homeobox 1	1

SMAD3	SMAD family member 3	1

KLF4	Kruppel-like factor 4 (gut)	1

KLF5	Kruppel-like factor 5 (intestinal)	1

CTBP2	C-terminal binding protein 2	1

GLI2	GLI family zinc finger 2	1

SQSTM1	sequestosome 1	2

ID1	inhibitor of DNA binding 1	2

HTATIP2	HIV-1 Tat interactive protein 2, 30kDa	2

SMARCC1	SWI/SNF related, matrix associated, actin dependent regulator of chromatin, subfamily c, member 1	2

ARID1A	AT rich interactive domain 1A (SWI-like)	2

MSX2	msh homeobox 2	2

IFI16	interferon, gamma-inducible protein 16	2

JARID2	AT rich interactive domain 2	2

E2F3	E2F transcription factor 3	2

IRF6	interferon regulatory factor 6	3

Table 1 lists the 18 transcription factors identified within the enriched sub-networks. The corresponding descriptions and locations within the sub-networks are included.

### The NRF2-mediated oxidative stress response pathway shows increased expression in arsenic-resistant tumor cell lines

We next set out to identify the canonical signaling pathways that possibly underlie tumor cell responses to arsenic trioxide by applying pathway analysis to the 209 differentially expressed genes (see Methods). A total of 177 genes were eligible for canonical pathway analysis.

**Figure 4:**
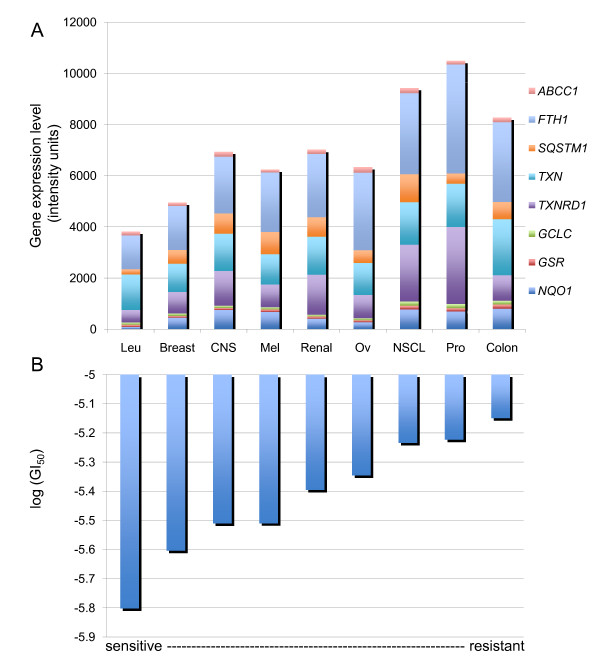
**Baseline expression levels of NRF2 target genes and tumor cell responses to arsenic trioxide**. (A) Baseline gene expression levels of eight NRF2 target genes in the NCI-60 tumor cell panel. For each of the nine tumor types, the average gene expression level was calculated for all the tumor cell lines within this group. The cumulative gene expression levels of the eight target genes were calculated to represent the general gene expression level of that tumor type (Leu = Leukemia; CNS = Central Nervous System; Mel = Melanoma; Ov = Ovarian; NSCL = Non-small Cell Lung; Pro = Prostate). (B) Arsenic-specific log(GI_50_) values of the NCI-60 tumor cell panel. For each of the nine tumor types, the average log(GI50) was calculated for all the tumor cell lines within this group to represent the general susceptibility of this tumor type to arsenic-induced cytotoxicity.

The NRF2-mediated oxidative stress response pathway was the most significant canonical pathway enriched in this dataset (p < 10^-3^). This canonical pathway contains eight NRF2 target genes, whose baseline expression levels were statistically associated with arsenic susceptibility (Figure 4). Interestingly, all eight genes showed high expression levels in the arsenic-resistant tumor cell lines (Figure 4). The eight genes are: ATP-binding cassette sub-family C (*CFTR/MRP*) member 1 (*ABCC1*), ferritin heavy polypeptide 1 (*FTH1*), glutamate-cysteine ligase catalytic subunit (*GCLC*), glutathione reductase (*GSR*), NAD(P)H dehydrogenase, quinone 1 (*NQO1*), sequestosome 1 (*SQSTM1*), thioredoxin (*TXN*), and thioredoxin reductase 1 (*TXNRD1*).

### Tumor cells deficient for NRF2 are sensitized to arsenic-induced cell killing

**Figure 5:**
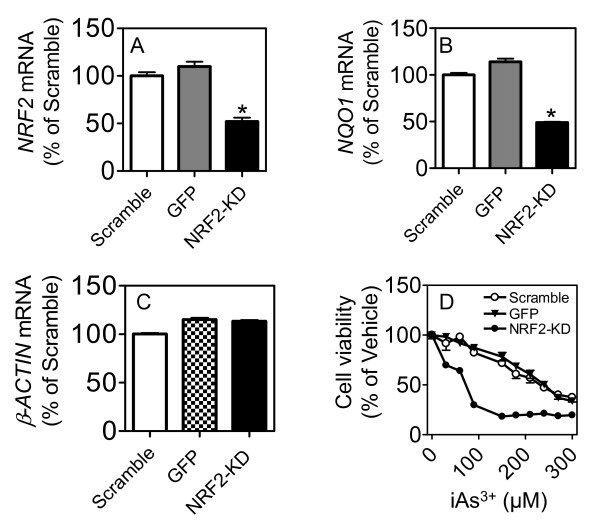
**Decreased NRF2 expression alters tumor cell response to arsenic trioxide**. Cells (A549 lung carcinoma) deficient for the expression of *NRF2 *(NRF2-KD), a scramble shRNA control (scramble), a turbo-GFP control (GFP) were generated using shRNAs and tested for their sensitivity to arsenic trioxide. (A) Cells expressing the NRF2-shRNA have decreased mRNA expression of *NRF2 *relative to the controls; (B) Cells expressing the NRF2-shRNA have decreased mRNA expression of *NQO1*, a well known NRF2 target gene; (C) Cells expressing the NRF2-shRNA show no alterations in mRNA expression of *β-ACTIN*; (D) Cells with decreased expression levels of NRF2 show increased sensitivity to arsenic trioxide induced killing.

To validate the role of NRF2 in mediating cellular survival in response to arsenic treatment, we generated tumor cells (A549 lung carcinoma) that were deficient for *NRF2 *expression using shRNAs (see Methods). As controls, we also generated tumor cells that expressed a scrambled shRNA sequence that activates the RNAi machinery without inducing knock-down of NRF2. Cells were exposed to arsenic trioxide over a dose range and their differential survival assessed after 24 hours. The data demonstrate that cells that are deficient for NRF2 are sensitized to arsenic-induced killing (Figure 5; Additional File 6).

## Discussion

In this study, we set out to identify gene biomarkers of tumor cell responses to arsenic trioxide-induced cytotoxicity. Using the cytotoxicity data established by the Developmental Therapeutics Program of the NCI, we ranked the tumor cell lines of the NCI-60 panel by their susceptibility to arsenic trioxide-induced killing. Through this ranking we find that there is a general trend of tumor cell susceptibility to arsenic trioxide for different tumor types. For instance, leukemia cell lines are distributed in the range of sensitivity to arsenic trioxide relative to the other tumor types. By associating the baseline gene expression levels of the NCI-60 human tumor cell panel with the arsenic trioxide-specific drug screening results, we identified 209 potential gene biomarkers with baseline expression levels that were significantly associated with tumor cell susceptibility to arsenic trioxide. Of the 209 genes, 169 (80.9%) were associated with arsenic resistance whereas the other 40 (19.1%) were associated with arsenic sensitivity. As expected, there is an association of the gene expression levels of these 209 genes with tumor type whereby many of same types of tumors show similar patterns of gene expression. As an example, in these analyses it is evident that the baseline gene expression levels of leukemia tumor cells with sensitivity to arsenic-induced killing are similar and cluster together. Likewise, colon tumor cells that show resistance to arsenic-induced killing also show baseline gene expression levels that are similar to each other, yet quite distinct from the leukemia tumor cell lines.

We applied a systems biology approach to examine these differentially expressed genes and affiliated networks and pathways, as well as the biological processes underlying tumor cell responses to arsenic-induced cytotoxicity. More specifically, in order to establish the potential biological mechanisms that underlie tumor cell responses to arsenic trioxide, we analyzed the 209 genes for known protein-protein interactions and enriched biological functions. We identified 64 common biological functions that were related to tumor cell responses to arsenic trioxide. Not surprisingly, we found that genes that are associated with arsenic susceptibility in the NCI-60 panel are statistically enriched for biological functions related to tumorigenesis, including cancer, cell death, cell-to-cell signaling and interaction, tumor morphology, and other functions relating to cancer disease.

We were intrigued to find numerous transcription factors with known links to tumorigenesis as well as with known association to arsenic trioxide are among our most significant arsenic-susceptibility gene biomarkers. For example, the transcription factor ID1 is well known for its function in carcinogenesis [25,28]. Furthermore, a study has shown that the ID1 was induced by inorganic arsenite and may contribute to cell survival after exposure to sodium arsenite [29]. Our findings suggest a potential link between the expression level of this transcription factor and how tumor cells respond when exposed to arsenic trioxide.

By examining canonical pathways in the gene biomarkers, we identified the enrichment of the NRF2-mediated oxidative stress response pathway. Specifically, eight NRF2 target genes were identified as significantly associated and all eight target genes showed high expression in arsenic-resistant tumor cell lines. The *NRF2 *gene itself did not show an association of its baseline gene expression and arsenic susceptibility. These findings may indicate that the arsenic-resistant tumor cell lines express the same levels of NRF2 mRNA but with higher transcriptional activity compared to the arsenic sensitive cell lines.

NRF2 is a transcription factor that responses to environmental hazardous insults [30], including reactive oxygen species (ROS) [31]. It has been a promising therapeutic target for various diseases [32-35] and recently linked to chemoprevention as well [14,36,37]. NRF2 works as a system with the protein Kelch-like ECH-associated protein 1 (KEAP1) [30]. Under normal conditions, NRF2 is bound by KEAP1 [38]. Exposure to NRF2 inducing agents results in the dissociation of NRF2 from KEAP1 and allows nuclear accumulation of NRF2, which triggers the expression of downstream target genes of NRF2 [30]. The NRF2 signaling pathway has been related to cell survival [39] and previous studies shown that NRF2 deficiency was associated with decreased rates on cell proliferation and tumor formation [40]. Interestingly, it has also been found that NRF2 and some of its downstream target genes were overexpressed in numerous tumor cell lines and human cancer tissues, which indicates its involvement in tumor formation [41-43]. NRF2 has also been shown to play a role in cellular responses to arsenic. For example, arsenic enhances the cellular expression of NRF2 at the transcript and protein levels and activates the expression of NRF2-related genes in skin cells [44]. In addition, arsenic-induced malignant transformation of human keratinocytes appears to require constitutive NRF2 activation [45].

To validate our computational prediction that NRF2 may mediate tumor cell survival in response to arsenic, we generated lung carcinoma cells that were deficient for the expression of NRF2. Through the computational analyses we predicted that cells with lower levels of NRF2 would be more sensitive to arsenic trioxide-induced killing. The results of the knock-down experiments support this and show that, as expected, cells that are deficient for NRF2 show increased sensitivity to arsenic-induced cytotoxicity. It should be noted the lung carcinoma cells that were used for these experiments are among the most resistant tumor cells of the NCI-60 panel to arsenic trioxide. It is therefore noteworthy that these highly resistant tumor cells can be altered to show increased cell killing to arsenic trioxide via their expression levels of NRF2.

Several of the NRF2 target genes identified from our study are of interest and support our findings in this work. For example, *TXN *and *TXNRD1 *are the key components of the thioredoxin system [46], which is an anti-oxidant system that has been linked to redoxinduced cell death [47], cellular growth [48], and apoptosis [49]. Previous studies shown that the redox status of *TXN *determines the sensitivity of human liver carcinoma cells (HepG2) to arsenic trioxide-induced cell death [50]. Moreover, research indicates that targeting the thioredoxin system to induce tumor cell apoptosis might underlie the anti-cancer mechanisms of several therapeutic agents, including arsenic trioxide [49].

*ABCC1 *is another noteworthy NRF2 target gene, and it is also known as multidrug resistance-associated protein 1 (MRP1). ABCC1 has been associated with chemotherapeutic resistance in several types of cancer [51], including cancers of the kidney [52], breast [53], and prostate [54,55]. ABCC1, as an ATP binding cassette protein, is believed to participate in chemotherapeutic agents transportation [51], including arsenic trioxide [56]; and possibly contributes to the chemoresistance in cancer treatment [51,57]. Chemotherapy resistance has been a huge obstacle in cancer treatment, and multidrug transporters like ABCC1 provide promising targets in chemotherapy [58-60] and valuable information for drug development. Our results indicate that *ABCC1 *could be a gene biomarker of arsenic response, as well as a potential chemotherapeutic target when using arsenic trioxide in cancer treatment, for APL and possibly other tumor types.

Another interesting finding is the identification of the transcription factor NFκB as an integrated node in the arsenic-susceptibility sub-network. NFκB is well known for its function in regulating genes for immune response, inflammation and apoptosis [61-63]. Numerous studies have shown that the NFκB signaling pathway is altered in the presence of arsenic trioxide [64-66]. For example, NFκB has been shown to be activated by arsenic at environmentally relevant concentrations [64,67-71] (reviewed in [72-74]). At higher doses, arsenic represses NF-κB activation [75]. The varied responses of NF-κB upon exposure to arsenic are certainly influenced by arsenic dose, arsenic species, and cell type differences. Similar to NRF2, the baseline expression levels of NFκB were not statistically associated with tumor cell responses to arsenic trioxide. However, its transcriptional targets are. Previous studies have demonstrated the crosstalk between NRF2 and NFκB in biological processes including inflammation and carcinogenesis [76,77], but the interaction between these two transcription factors under cellular stress is not clearly understood. Our results suggest that NRF2 and NFκB both may contribute to tumor cell resistance upon exposure to arsenic trioxide, and the two transcription factors may work cooperatively in protecting tumor cells from arsenic-induced cytotoxicity.

## Conclusions

In this study, we identified potential gene biomarkers of tumor cell responses to arsenic trioxide. These gene biomarkers have baseline expression levels that are statistically associated with tumor cell susceptibility to arsenic trioxide. Among the biomarkers are genes that are enriched for the NRF2 pathway. Using shRNA-mediated knock-down in a highly resistant lung tumor cell line, we show for the first time that deficiency for NRF2 in a tumor cell line results in increased sensitivity to arsenic trioxide. It may be the case that the other gene biomarkers are also potential modulators of cellular response to arsenic-induced cytotoxicity. The identification of the genetic factors such as NRF2 that underlie the tumor cell responses to arsenic trioxide will have direct implications in the continued application of arsenic trioxide as a chemotherapeutic agent in treating APL and other types of cancer. For example, these results can be applied for a better understanding of which tumor types will be responsive to arsenic treatment, thus facilitating the development of personalized medication.

## List of abbreviations

APL: acute promyelocytic leukemia; GI50: Growth Inhibition of 50%; DTP: Developmental Therapeutics Program; NRF2: nuclear factor erythroid 2-related factor 2; WHO World Health Organization; EPA: US Environmental Protection Agency; ppb - parts per billion; ATRA: all-trans-retinoic acid; NCI - national cancer institute; Bcl-2: B-cell CLL/lymphoma 2; NFκB: Nuclear Factor kappa B; SAM - Significance Analysis of Microarrays; FDR - False Discovery Rate; ELF3: E74-like factor 3; MTF2: metal response element binding transcription factor 2; ZEB1: zinc finger E-box binding homeobox 1; SMAD3: SMAD family member 3; KLF4: Kruppel-like factor 4 (gut); KLF5: Kruppel-like factor 5 (intestinal); CTBP2: C-terminal binding protein 2; GLI2 GLI family zinc finger 2; SQSTM1: sequestosome 1; ID1: inhibitor of DNA binding 1; HTATIP2: HIV-1 Tat interactive protein 2, 30kDa; SMARCC1: SWI/SNF related, matrix associated, actin dependent regulator of chromatin, subfamily c, member 1; ARID1A: AT rich interactive domain 1A (SWI-like); MSX2: msh homeobox 2; IFI16 interferon, gamma-inducible protein 16; JARID2: jumonji, AT rich interactive domain 2; E2F3: E2F transcription factor 3; IRF6: interferon regulatory factor 6; Ap1 Activator Protein 1; ABCC1: ATP-binding cassette sub-family C (CFTR/MRP) member 1; FTH1: ferritin heavy polypeptide 1; GCLC: glutamate-cysteine ligase catalytic subunit; GSR: glutathione reductase; NQO1: NAD(P)H dehydrogenase, quinone 1; TXN - thioredoxin; TXNRD1: thioredoxin reductase 1; RAS: Reactive Oxygen Species; Keap1: Kelch-like ECH-associated protein 1; MRP1: Multidrug Resistance-associated Protein 1; IVCLSP: *In Vitro *Cell Line Screening Project; shRNAs-short hairpin RNAs

## Competing interests

The authors declare that they have no competing interests.

## Authors' contributions

QL performed the computational analysis and drafted the manuscript. HZ generated knock-down cells, performed cell killing assays and real time PCR. LS performed arsenic-trioxide killing assays and assisted with manuscript preparation. FZ assisted with data analysis and interpretation. MK assisted with LDH assays. IJ assisted with manuscript preparation and oversaw LDH assays. JP oversaw knock-down experiments, RT-PCR analysis, killing assays and assisted with manuscript preparation. RF conceived and designed experiments, assisted with computational analysis, and wrote the manuscript. All authors read and approved the final manuscript.

## Pre-publication history

The pre-publication history for this paper can be accessed here:

http://www.biomedcentral.com/1755-8794/3/37/prepub

## Supplementary Material

Additional file 1 **GI_50 _of 59 cell lines of the NCI-60 human tumor cell panel**. Lists the GI50 of 59 cell lines of the NCI-60 cell panel. Corresponding tumor type, cell line number, and cell line name are included. The cell lines were numbered from 1 to 59, according to cellular sensitivity to arsenic trioxide (e.g. cell line number 1 is the most resistant cell line, whereas cell line 59 is the most sensitive cell line.) Click here for file

Additional file 2 **Potential gene biomarkers of tumor cell susceptibility to arsenic trioxide**. Lists all the 242 gene probes (209 genes) that were statistically associated with tumor cell susceptibility to arsenic trioxide. Corresponding gene IDs, q-values, and gene descriptions are included. Click here for file

Additional file 3 **Gene products in network analysis**. Lists all the 317 proteins contained within the large interactome. Each protein is listed as either its baseline expression level statistically associated with arsenic resistance/sensitivity, or it interacts with the directly associated transcripts. Corresponding gene symbols, gene names, gene IDs, and other relative information are included. Proteins within the three most significant sub-networks are also identified. Click here for file

Additional file 4 **64 common biological functions enriched in arsenic susceptibility associated networks**. Lists all the 64 biological functions enriched in arsenic susceptibility associated networks. The functional category, p-value, and arsenic susceptibility-associated molecules within these functions are included. Click here for file

Additional file 5 **Ten sub-networks within the large interactome**. Lists the top ten sub-networks within the large interactome. Networks were built on the "Focus Molecules", whose baseline expression levels are statistically associated with tumor cell susceptibility to arsenic trioxide. Molecules in the networks are either focus molecules (e.g. gene biomarkers of arsenic susceptibility) or molecules interact with them. P-values for the ten sub-networks are detailed. Click here for file

Additional file 6 **LDH release in NRF2 knock-down tumor cells**. Lactate dehydrogenase (LDH) release was measured in NRF2 knock-down tumor (A549) cells (NRF2-KD) or control cells (expressing GFP reporter) exposed to inorganic arsenic.* indicates p < 0.05. Click here for file
