# Supplementary material for: The NRF2-mediated oxidative stress response pathway is associated with tumor cell resistance to arsenic trioxide across the NCI-60 panel
Source: BMC Med Genomics. 2010 Aug 13;3:37. doi: 10.1186/1755-8794-3-37 (PMC2939609; doi:10.1186/1755-8794-3-37)
Supplement: Additional file 1 — GI50 of 59 cell lines of the NCI-60 human tumor cell panel. Lists the GI50 of 59 cell lines of the NCI-60 cell panel. Corresponding tumor type, cell line number, and cell line name are included. The cell lines were numbered from 1 to 59, according to cellular sensitivity to arsenic trioxide (e.g. cell line number 1 is the most resistant cell line, whereas cell line 59 is the most sensitive cell line.) [file 1755-8794-3-37-S1.PDF]

Additional File 1: NCI-60 tumor cell lines

Cell Line Number	Tumor type	Cell Line Name	-Log(GI_{50})
1	Colon	HCC-2998	4.776
2	Non-small Cell Lung	A549/ATCC	4.837
3	Colon	KM12	4.847
4	Non-small Cell Lung	NCI-H322M	4.887
5	Central Nervous System	SF-295	4.93
6	Ovarian	SK-OV-3	4.96
7	Renal	TK-10	5.037
8	Prostate	DU-145	5.054
9	Renal	ACHN	5.055
10	Non-small Cell Lung	NCI-H226	5.075
11	Colon	COLO 205	5.104
12	Colon	HCT-116	5.113
13	Melanoma	MDA-MB-435	5.132
14	Non-small Cell Lung	NCI-H460	5.132
15	Ovarian	OVCAR-5	5.186
16	Non-small Cell Lung	HOP-62	5.198
17	Ovarian	NCI/ADR-RES	5.207
18	Renal	A498	5.267
19	Ovarian	OVCAR-4	5.322
20	Colon	HT29	5.346
21	Melanoma	SK-MEL-28	5.377
22	Breast	MDA-MB-231/ATCC	5.381
23	Prostate	PC-3	5.392
24	Renal	UO-31	5.394
25	Melanoma	UACC-257	5.4
26	Central Nervous System	SNB-19	5.405
27	Colon	HCT-15	5.419
28	Colon	SW-620	5.441
29	Non-small Cell Lung	EKVX	5.451
30	Ovarian	OVCAR-8	5.46
31	Breast	MDA-N	5.467
32	Melanoma	M14	5.469
33	Renal	SN12C	5.476
34	Non-small Cell Lung	HOP-92	5.508
35	Melanoma	UACC-62	5.529
36	Non-small Cell Lung	NCI-H23	5.535
37	Renal	RXF 393	5.543
38	Ovarian	OVCAR-3	5.581
39	Breast	MCF7	5.582

Additional File 1: NCI-60 tumor cell lines

**40**	Renal	786-0	5.588
**41**	Leukemia	HL-60(TB)	5.62
**42**	Melanoma	SK-MEL-2	5.621
**43**	Central Nervous System	SF-539	5.63
**44**	Melanoma	SK-MEL-5	5.638
**45**	Central Nervous System	SF-268	5.643
**46**	Leukemia	MOLT-4	5.648
**47**	Breast	HS 578T	5.652
**48**	Central Nervous System	SNB-75	5.677
**49**	Melanoma	LOX IMVI	5.69
**50**	Leukemia	RPMI-8226	5.699
**51**	Ovarian	IGROV1	5.703
**52**	Leukemia	K-562	5.724
**53**	Melanoma	MALME-3M	5.733
**54**	Central Nervous System	U251	5.777
**55**	Non-small Cell Lung	NCI-H522	5.786
**56**	Renal	CAKI-1	5.804
**57**	Leukemia	SR	5.867
**58**	Breast	BT-549	5.937
**59**	Leukemia	CCRF-CEM	6.254
