# Supplementary material for: The NRF2-mediated oxidative stress response pathway is associated with tumor cell resistance to arsenic trioxide across the NCI-60 panel
Source: BMC Med Genomics. 2010 Aug 13;3:37. doi: 10.1186/1755-8794-3-37 (PMC2939609; doi:10.1186/1755-8794-3-37)
Supplement: Additional file 2 — Potential gene biomarkers of tumor cell susceptibility to arsenic trioxide. Lists all the 242 gene probes (209 genes) that were statistically associated with tumor cell susceptibility to arsenic trioxide. Corresponding gene IDs, q-values, and gene descriptions are included. [file 1755-8794-3-37-S2.PDF]

Additional File 2: potential gene biomarkers

No.	Association	Gene ID	q-value (%)	Gene Symbol	Gene Description
1	sensitivity	212738_at	5.28	ARHGAP19	Rho GTPase activating protein 19
2	sensitivity	212919_at	5.28	DCP2	DCP2 decapping enzyme homolog (S. cerevisiae)
3	sensitivity	203297_s_at	5.28	JARID2	jumonji, AT rich interactive domain 2
4	sensitivity	218733_at	5.28	MSL2L1	male-specific lethal 2-like 1 (Drosophila)
5	sensitivity	201074_at	5.28	SMARCC1	SWI/SNF related, matrix associated, actin dependent regulator of chromatin, subfamily c, member 1
6	sensitivity	217812_at	5.28	YTHDF2	YTH domain family, member 2
7	sensitivity	200783_s_at	5.28	STMN1	stathmin 1/oncoprotein 18
8	sensitivity	212758_s_at	5.28	ZEB1	zinc finger E-box binding homeobox 1
9	sensitivity	209240_at	5.28	OGT	O-linked N-acetylglucosamine (GlcNAc) transferase (UDP-N-acetylglucosamine:polypeptide-N-acetylglucosaminyl transferase)

10	sensitivity	203693_s_at	5.28	E2F3	E2F transcription factor 3
11	sensitivity	202602_s_at	5.28	HTATSF1	HIV-1 Tat specific factor 1
12	sensitivity	203298_s_at	5.28	JARID2	jumonji, AT rich interactive domain 2
13	sensitivity	208965_s_at	5.28	IFI16	interferon, gamma-inducible protein 16
14	sensitivity	211987_at	5.28	TOP2B	topoisomerase (DNA) II beta 180kDa
15	sensitivity	206554_x_at	5.28	SETMAR	SET domain and mariner transposase fusion gene
16	sensitivity	220079_s_at	5.28	USP48	ubiquitin specific peptidase 48
17	sensitivity	205739_x_at	5.28	ZNF107	zinc finger protein 107
18	sensitivity	218600_at	5.28	LIMD2	LIM domain containing 2
19	sensitivity	209704_at	5.28	MTF2	metal response element binding transcription factor 2

Additional File 2: potential gene biomarkers

20	sensitivity	204795_at	5.28	PRR3	proline rich 3
21	sensitivity	214988_s_at	5.28	SON	SON DNA binding protein
22	sensitivity	201312_s_at	5.28	SH3BGRL	SH3 domain binding glutamic acid-rich protein like
23	sensitivity	213301_x_at	5.28	TRIM24	tripartite motif-containing 24
24	sensitivity	203316_s_at	5.28	SNRPE	small nuclear ribonucleoprotein polypeptide E
25	sensitivity	214093_s_at	5.28	FUBP1	far upstream element (FUSE) binding protein 1
26	sensitivity	208668_x_at	5.28	HMGN2	high-mobility group nucleosomal binding domain 2
27	sensitivity	218223_s_at	5.28	PLEKHO1	pleckstrin homology domain containing, family O member 1
28	sensitivity	212605_s_at	5.28	---	CDNA FLJ32526 fis, clone SMINT2000073
29	sensitivity	221912_s_at	5.28	CCDC28B	coiled-coil domain containing 28B

Additional File 2: potential gene biomarkers

30	sensitivity	209033_s_at	5.28	DYRK1A	dual-specificity tyrosine-(Y)-phosphorylation regulated kinase 1A
31	sensitivity	214937_x_at	5.28	PCM1	pericentriolar material 1
32	sensitivity	209705_at	5.28	MTF2	metal response element binding transcription factor 2
33	sensitivity	212880_at	5.28	WDR7	WD repeat domain 7
34	sensitivity	203346_s_at	5.28	MTF2	metal response element binding transcription factor 2
35	sensitivity	210649_s_at	5.28	ARID1A	AT rich interactive domain 1A (SWI-like)
36	sensitivity	212847_at	5.28	FUBP1	Far upstream element (FUSE) binding protein 1
37	sensitivity	211946_s_at	5.28	BAT2D1	BAT2 domain containing 1
38	sensitivity	221230_s_at	5.28	ARID4B	AT rich interactive domain 4B (RBP1-like)
39	sensitivity	221952_x_at	5.28	TRMT5	TRM5 tRNA methyltransferase 5 homolog (S. cerevisiae)

Additional File 2: potential gene biomarkers

40	sensitivity	212587_s_at	5.28	PTPRC	protein tyrosine phosphatase, receptor type, C
41	sensitivity	201969_at	5.28	NASP	nuclear autoantigenic sperm protein (histone-binding)
42	sensitivity	212414_s_at	5.28	N-PAC /// SEPT6	septin 6 /// cytokine-like nuclear factor n-pac
43	sensitivity	214016_s_at	5.28	SFPQ	splicing factor proline/glutamine-rich (polypyrimidine tract binding protein associated)
44	sensitivity	218437_s_at	5.28	LZTFL1	leucine zipper transcription factor-like 1
45	resistance	209529_at	0.00	PPAP2C	phosphatidic acid phosphatase type 2C
46	resistance	207180_s_at	0.00	HTATIP2	HIV-1 Tat interactive protein 2, 30kDa
47	resistance	33323_r_at	0.00	SFN	stratifin
48	resistance	33322_i_at	0.00	SFN	stratifin
49	resistance	201650_at	0.00	KRT19	keratin 19

50	resistance	209260_at	0.00	SFN	stratifin
51	resistance	209448_at	0.00	HTATIP2	HIV-1 Tat interactive protein 2, 30kDa
52	resistance	209008_x_at	0.00	KRT8	keratin 8
53	resistance	212925_at	0.00	C19orf21	chromosome 19 open reading frame 21
54	resistance	202923_s_at	0.00	GCLC	glutamate-cysteine ligase, catalytic subunit
55	resistance	200001_at	0.00	CAPNS1	calpain, small subunit 1
56	resistance	202922_at	0.00	GCLC	glutamate-cysteine ligase, catalytic subunit
57	resistance	219429_at	0.00	FA2H	fatty acid 2-hydroxylase
58	resistance	202054_s_at	0.00	ALDH3A2	aldehyde dehydrogenase 3 family, member A2
59	resistance	204059_s_at	0.00	ME1	malic enzyme 1, NADP(+)-dependent, cytosolic

Additional File 2: potential gene biomarkers

60	resistance	201596_x_at	0.00	KRT18	keratin 18
61	resistance	32837_at	0.00	AGPAT2	1-acylglycerol-3-phosphate O-acyltransferase 2 (lysophosphatidic acid acyltransferase, beta)
62	resistance	217000_at	0.00	KRT18P50	keratin 18 pseudogene 50
63	resistance	210827_s_at	0.00	ELF3	E74-like factor 3 (ets domain transcription factor, epithelial-specific)
64	resistance	204034_at	0.00	ETHE1	ethylmalonic encephalopathy 1
65	resistance	220189_s_at	0.00	MGAT4B	mannosyl (alpha-1,3-)-glycoprotein beta-1,4-N-acetylglucosaminyltransferase, isozyme B
66	resistance	210519_s_at	0.00	NQO1	NAD(P)H dehydrogenase, quinone 1
67	resistance	212823_s_at	0.00	PLEKHG3	pleckstrin homology domain containing, family G (with RhoGef domain) member 3
68	resistance	202053_s_at	0.00	ALDH3A2	aldehyde dehydrogenase 3 family, member A2
69	resistance	218035_s_at	0.00	RBM47	RNA binding motif protein 47

Additional File 2: potential gene biomarkers

70	resistance	217351_at	0.00	---	---
71	resistance	211653_x_at	0.00	AKR1C2	aldo-keto reductase family 1, member C2 (dihydrodiol dehydrogenase 2; bile acid binding protein; 3-alpha hydroxysteroid dehydrogenase, type III)
72	resistance	201467_s_at	0.00	NQO1	NAD(P)H dehydrogenase, quinone 1
73	resistance	208613_s_at	0.00	FLNB	filamin B, beta (actin binding protein 278)
74	resistance	205770_at	0.00	GSR	glutathione reductase
75	resistance	209873_s_at	0.00	PKP3	plakophilin 3
76	resistance	203108_at	0.00	GPRC5A	G protein-coupled receptor, family C, group 5, member A
77	resistance	208161_s_at	0.00	ABCC3	ATP-binding cassette, sub-family C (CFTR/MRP), member 3
78	resistance	207528_s_at	0.00	SLC7A11	solute carrier family 7, (cationic amino acid transporter, y+ system) member 11
79	resistance	212444_at	0.00	---	CDNA clone IMAGE:6025865

80	resistance	209699_x_at	0.00	AKR1C2	aldo-keto reductase family 1, member C2 (dihydrodiol dehydrogenase 2; bile acid binding protein; 3-alpha hydroxysteroid dehydrogenase, type III)
81	resistance	200872_at	0.00	S100A10	S100 calcium binding protein A10
82	resistance	204151_x_at	0.00	AKR1C1	aldo-keto reductase family 1, member C1 (dihydrodiol dehydrogenase 1; 20-alpha (3-alpha)-hydroxysteroid dehydrogenase)
83	resistance	219045_at	0.00	RHOF	ras homolog gene family, member F (in filopodia)
84	resistance	218511_s_at	0.00	PNPO	pyridoxamine 5-phosphate oxidase
85	resistance	205093_at	0.00	PLEKHA6	pleckstrin homology domain containing, family A member 6
86	resistance	216594_x_at	0.00	AKR1C1	aldo-keto reductase family 1, member C1 (dihydrodiol dehydrogenase 1; 20-alpha (3-alpha)-hydroxysteroid dehydrogenase)
87	resistance	201468_s_at	0.00	NQO1	NAD(P)H dehydrogenase, quinone 1
88	resistance	209373_at	0.00	MALL	mal, T-cell differentiation protein-like
89	resistance	217901_at	0.00	DSG2	Desmoglein 2

Additional File 2: potential gene biomarkers

90	resistance	205015_s_at	0.00	TGFA	transforming growth factor, alpha
91	resistance	204990_s_at	0.00	ITGB4	integrin, beta 4
92	resistance	209641_s_at	0.00	ABCC3	ATP-binding cassette, sub-family C (CFTR/MRP), member 3
93	resistance	209607_x_at	0.77	GIYD1 /// GIYD2 /// SULT1A3 /// SULT1A4	sulfotransferase family, cytosolic, 1A, phenol-preferring, member 3 GIY-YIG domain containing 2 sulfotransferase family, cytosolic, 1A, phenol-preferring, member 4 GIY-YIG domain containing 1
94	resistance	210544_s_at	0.77	ALDH3A2	aldehyde dehydrogenase 3 family, member A2
95	resistance	221566_s_at	0.77	NOL3	nucleolar protein 3 (apoptosis repressor with CARD domain)
96	resistance	222125_s_at	0.77	PH-4	hypoxia-inducible factor prolyl 4-hydroxylase
97	resistance	214676_x_at	0.77	MUC3A	mucin 3A, cell surface associated
98	resistance	210678_s_at	0.77	AGPAT2	1-acylglycerol-3-phosphate O-acyltransferase 2 (lysophosphatidic acid acyltransferase, beta)
99	resistance	209921_at	0.77	SLC7A11	solute carrier family 7, (cationic amino acid transporter, y+ system) member 11

100	resistance	214211_at	0.77	FTH1	ferritin, heavy polypeptide 1
101	resistance	209160_at	0.77	AKR1C3	aldo-keto reductase family 1, member C3 (3-alpha hydroxysteroid dehydrogenase, type II)
102	resistance	205490_x_at	0.77	GJB3	gap junction protein, beta 3, 31kDa
103	resistance	202794_at	0.77	INPP1	inositol polyphosphate-1-phosphatase
104	resistance	210896_s_at	1.31	ASPH	aspartate beta-hydroxylase
105	resistance	213590_at	1.31	LOC100133772 /// SLC16A5	solute carrier family 16, member 5 (monocarboxylic acid transporter 6) similar to MCT
106	resistance	201266_at	1.31	TXNRD1	thioredoxin reductase 1
107	resistance	216821_at	1.31	KRT8 /// KRT8P9 /// LOC149501 /// LOC647954	keratin 8 /// similar to keratin 8 /// keratin 8 pseudogene 9
108	resistance	212314_at	1.31	KIAA0746 /// SERINC2	KIAA0746 protein /// serine incorporator 2
109	resistance	202593_s_at	1.31	GDE1	glycerophosphodiester phosphodiesterase 1

110	resistance	211628_x_at	1.31	FTHP1	ferritin, heavy polypeptide pseudogene 1
111	resistance	205145_s_at	1.31	LOC649851 /// MYL5	myosin, light chain 5, regulatory /// similar to Superfast myosin regulatory light chain 2 (MyLC-2) (MYLC2) (Myosin regulatory light chain 5)
112	resistance	215243_s_at	1.31	GJB3	gap junction protein, beta 3, 31kDa
113	resistance	218966_at	1.31	MYO5C	myosin VC
114	resistance	218796_at	2.08	FERMT1	fermitin family homolog 1 (Drosophila)
115	resistance	206354_at	2.08	LST-3TM12 /// SLCO1B1 /// SLCO1B3	solute carrier organic anion transporter family, member 1B1 /// solute carrier organic anion transporter family, member 1B3 /// organic anion transporter LST-3b
116	resistance	220073_s_at	2.08	PLEKHG6	pleckstrin homology domain containing, family G (with RhoGef domain) member 6
117	resistance	200748_s_at	2.08	FTH1	ferritin, heavy polypeptide 1
118	resistance	205455_at	2.08	MST1R	macrophage stimulating 1 receptor (c-met-related tyrosine kinase)
119	resistance	206723_s_at	2.08	LPAR2	lysophosphatidic acid receptor 2

120	resistance	208526_at	2.08	OR2F1	olfactory receptor, family 2, subfamily F, member 1
121	resistance	219150_s_at	2.08	CENTA1	centaurin, alpha 1
122	resistance	208614_s_at	2.08	FLNB	filamin B, beta (actin binding protein 278)
123	resistance	222349_x_at	2.08	RNF126P1	ring finger protein 126 pseudogene 1
124	resistance	210580_x_at	2.08	GIYD1 /// GIYD2 /// SULT1A3 /// SULT1A4	sulfotransferase family, cytosolic, 1A, phenol-preferring, member 3 GIY-YIG domain containing 2 sulfotransferase family, cytosolic, 1A, phenol-preferring, member 4 GIY-YIG domain containing 1
125	resistance	218644_at	2.08	PLEK2	pleckstrin 2
126	resistance	208862_s_at	2.08	CTNND1	catenin (cadherin-associated protein), delta 1
127	resistance	208864_s_at	2.08	TXN	thioredoxin
128	resistance	204856_at	2.08	B3GNT3	UDP-GlcNAc:betaGal beta-1,3-N-acetylglucosaminyltransferase 3
129	resistance	202804_at	2.08	ABCC1	ATP-binding cassette, sub-family C (CFTR/MRP), member 1

Additional File 2: potential gene biomarkers

130	resistance	215715_at	2.08	SLC6A2	solute carrier family 6 (neurotransmitter transporter, noradrenalin), member 2
131	resistance	210177_at	2.08	TRIM15	tripartite motif-containing 15
132	resistance	209173_at	3.17	AGR2	anterior gradient homolog 2 (Xenopus laevis)
133	resistance	210554_s_at	3.17	CTBP2	C-terminal binding protein 2
134	resistance	205640_at	3.17	ALDH3B1	aldehyde dehydrogenase 3 family, member B1
135	resistance	221232_s_at	3.17	ANKRD2	ankyrin repeat domain 2 (stretch responsive muscle)
136	resistance	201471_s_at	3.17	SQSTM1	sequestosome 1
137	resistance	204608_at	3.17	ASL	argininosuccinate lyase
138	resistance	208771_s_at	3.17	LTA4H	leukotriene A4 hydrolase
139	resistance	201220_x_at	3.17	CTBP2	C-terminal binding protein 2

140	resistance	209759_s_at	3.17	DCI	dodecenoyl-Coenzyme A delta isomerase (3,2 trans-enoyl-Coenzyme A isomerase)
141	resistance	204519_s_at	3.17	PLLP	plasma membrane proteolipid (plasmolipin)
142	resistance	209275_s_at	3.17	CLN3	ceroid-lipofuscinosis, neuronal 3, juvenile (Batten, Spielmeyer-Vogt disease)
143	resistance	201287_s_at	3.17	SDC1	syndecan 1
144	resistance	210761_s_at	3.17	GRB7	growth factor receptor-bound protein 7
145	resistance	204503_at	3.17	EVPL	envoplakin
146	resistance	217678_at	3.17	SLC7A11	solute carrier family 7, (cationic amino acid transporter, y+ system) member 11
147	resistance	219513_s_at	3.17	SH2D3A	SH2 domain containing 3A
148	resistance	202267_at	3.17	LAMC2	laminin, gamma 2
149	resistance	205074_at	3.17	SLC22A5	solute carrier family 22 (organic cation/carnitine transporter), member 5

150	resistance	205555_s_at	3.17	MSX2	msh homeobox 2
151	resistance	209212_s_at	3.17	KLF5	Kruppel-like factor 5 (intestinal)
152	resistance	201463_s_at	3.17	LOC100133665 /// TALDO1	transaldolase 1 /// similar to transaldolase
153	resistance	201243_s_at	3.17	ATP1B1	ATPase, Na+/K+ transporting, beta 1 polypeptide
154	resistance	207034_s_at	3.17	GLI2	GLI-Kruppel family member GLI2
155	resistance	204058_at	3.17	ME1	malic enzyme 1, NADP(+)-dependent, cytosolic
156	resistance	212339_at	3.17	EPB41L1	erythrocyte membrane protein band 4.1-like 1
157	resistance	218900_at	3.17	CNNM4	cyclin M4
158	resistance	205397_x_at	3.17	SMAD3	SMAD family member 3
159	resistance	204751_x_at	3.17	DSC2	desmocollin 2

Additional File 2: potential gene biomarkers

160	resistance	218186_at	3.17	RAB25	RAB25, member RAS oncogene family
161	resistance	209558_s_at	3.17	HIP1R	huntingtin interacting protein 1 related
162	resistance	209135_at	3.17	ASPH	aspartate beta-hydroxylase
163	resistance	208510_s_at	3.17	PPARG	peroxisome proliferator-activated receptor gamma
164	resistance	211241_at	3.17	ANXA2P3	annexin A2 pseudogene 3
165	resistance	202204_s_at	3.17	AMFR	autocrine motility factor receptor
166	resistance	217149_x_at	3.17	TNK1	tyrosine kinase, non-receptor, 1
167	resistance	222060_at	3.17	KRT8P12	keratin 8 pseudogene 12
168	resistance	202790_at	3.17	CLDN7	claudin 7
169	resistance	203458_at	3.17	SPR	sepiapterin reductase (7,8-dihydrobiopterin:NADP+ oxidoreductase)

170	resistance	90265_at	3.17	CENTA1	centaurin, alpha 1
171	resistance	204855_at	3.17	SERPINB5	serpin peptidase inhibitor, clade B (ovalbumin), member 5
172	resistance	219005_at	3.17	TMEM59L	transmembrane protein 59-like
173	resistance	204341_at	3.17	TRIM16	tripartite motif-containing 16
174	resistance	205623_at	4.65	ALDH3A1	aldehyde dehydrogenase 3 family, memberA1
175	resistance	209369_at	4.65	ANXA3	annexin A3
176	resistance	48106_at	4.65	FLJ20489	hypothetical protein FLJ20489
177	resistance	212311_at	4.65	KIAA0746 /// SERINC2	KIAA0746 protein /// serine incorporator 2
178	resistance	217730_at	4.65	TMBIM1	transmembrane BAX inhibitor motif containing 1
179	resistance	207684_at	4.65	TBX6	T-box 6

180	resistance	217939_s_at	4.65	AFTPH	aftiphilin
181	resistance	213085_s_at	4.65	WWC1	WW and C2 domain containing 1
182	resistance	221256_s_at	4.65	HDHD3	haloacid dehalogenase-like hydrolase domain containing 3
183	resistance	214898_x_at	4.65	MUC3B	mucin 3B, cell surface associated
184	resistance	218677_at	4.65	S100A14	S100 calcium binding protein A14
185	resistance	201118_at	4.65	PGD	phosphogluconate dehydrogenase
186	resistance	203955_at	4.65	KIAA0649	KIAA0649
187	resistance	202275_at	4.65	G6PD	glucose-6-phosphate dehydrogenase
188	resistance	201590_x_at	4.65	ANXA2	annexin A2
189	resistance	208190_s_at	4.65	LSR	lipolysis stimulated lipoprotein receptor

Additional File 2: potential gene biomarkers

190	resistance	201219_at	4.65	CTBP2	C-terminal binding protein 2
191	resistance	218164_at	4.65	SPATA20	spermatogenesis associated 20
192	resistance	219508_at	4.65	GCNT3	glucosaminyl (N-acetyl) transferase 3, mucin type
193	resistance	210876_at	4.65	ANXA2P1	annexin A2 pseudogene 1
194	resistance	222154_s_at	4.65	LOC26010	viral DNA polymerase-transactivated protein 6
195	resistance	204540_at	4.65	EEF1A2	eukaryotic translation elongation factor 1 alpha 2
196	resistance	217806_s_at	4.65	POLDIP2	polymerase (DNA-directed), delta interacting protein 2
197	resistance	209622_at	4.65	STK16	serine/threonine kinase 16
198	resistance	218245_at	4.65	TSKU	tsukushin
199	resistance	203557_s_at	4.65	PCBD1	pterin-4 alpha-carbinolamine dehydratase/dimerization cofactor of hepatocyte nuclear factor 1 alpha

Additional File 2: potential gene biomarkers

200	resistance	216641_s_at	4.65		ladinin 1
201	resistance	210835_s_at	4.65	CTBP2	C-terminal binding protein 2
202	resistance	201428_at	4.65	CLDN4	claudin 4
203	resistance	208937_s_at	4.65	ID1	inhibitor of DNA binding 1, dominant negative helix-loop-helix protein
204	resistance	201933_at	4.65	CHMP1A	chromatin modifying protein 1A
205	resistance	205398_s_at	4.65	SMAD3	SMAD family member 3
206	resistance	200965_s_at	4.65	ABLIM1	actin binding LIM protein 1
207	resistance	40359_at	4.65	RASSF7	Ras association (RalGDS/AF-6) domain family (N-terminal) member 7
208	resistance	221841_s_at	4.65	KLF4	Kruppel-like factor 4 (gut)
209	resistance	218622_at	4.65	NUP37	nucleoporin 37kDa

210	resistance	214088_s_at	4.65	FUT3	fucosyltransferase 3 (galactoside 3(4)-L-fucosyltransferase, Lewis blood group)
211	resistance	205536_at	4.65	VAV2	vav 2 guanine nucleotide exchange factor
212	resistance	213503_x_at	4.65	ANXA2	annexin A2
213	resistance	207525_s_at	4.65	GIPC1	GIPC PDZ domain containing family, member 1
214	resistance	217333_at	4.65	KRT18P44	keratin 18 pseudogene 44
215	resistance	206561_s_at	4.65	AKR1B10	aldo-keto reductase family 1, member B10 (aldose reductase)
216	resistance	205073_at	4.65	CYP2J2	cytochrome P450, family 2, subfamily J, polypeptide 2
217	resistance	215913_s_at	4.65	GULP1	GULP, engulfment adaptor PTB domain containing 1
218	resistance	205792_at	4.65	WISP2	WNT1 inducible signaling pathway protein 2
219	resistance	214647_s_at	4.65	HFE	hemochromatosis

Additional File 2: potential gene biomarkers

220	resistance	216074_x_at	4.65	WWC1	WW and C2 domain containing 1
221	resistance	205774_at	4.65	F12	coagulation factor XII (Hageman factor)
222	resistance	206429_at	4.65	F2RL1	coagulation factor II (thrombin) receptor-like 1
223	resistance	201839_s_at	4.65	TACSTD1	tumor-associated calcium signal transducer 1
224	resistance	219580_s_at	4.65	TMC5	transmembrane channel-like 5
225	resistance	31846_at	5.28	RHOD	ras homolog gene family, member D
226	resistance	221241_s_at	5.28	BCL2L14	BCL2-like 14 (apoptosis facilitator)
227	resistance	202671_s_at	5.28	PDXK	pyridoxal (pyridoxine, vitamin B6) kinase
228	resistance	206482_at	5.28	PTK6	PTK6 protein tyrosine kinase 6
229	resistance	208816_x_at	5.28	ANXA2P2	annexin A2 pseudogene 2

Additional File 2: potential gene biomarkers

230	resistance	203786_s_at	5.28	TPD52L1	tumor protein D52-like 1
231	resistance	210021_s_at	5.28	CCNO	cyclin O
232	resistance	205032_at	5.28	ITGA2	integrin, alpha 2 (CD49B, alpha 2 subunit of VLA-2 receptor)
233	resistance	201005_at	5.28	CD9	CD9 molecule
234	resistance	206600_s_at	5.28	LOC100133772 /// SLC16A5	solute carrier family 16, member 5 (monocarboxylic acid transporter 6) similar to MCT
235	resistance	204148_s_at	5.28	POMZP3 /// ZP3	zona pellucida glycoprotein 3 (sperm receptor) /// POM (POM121 homolog, rat) and ZP3 fusion
236	resistance	208890_s_at	5.28	PLXNB2	plexin B2
237	resistance	207368_at	5.28	HTR1D	5-hydroxytryptamine (serotonin) receptor 1D
238	resistance	201717_at	5.28	MRPL49	mitochondrial ribosomal protein L49
239	resistance	212093_s_at	5.28	MTUS1	mitochondrial tumor suppressor 1

240	resistance	202597_at	5.28	IRF6	interferon regulatory factor 6
241	resistance	208022_s_at	5.28	CDC14B /// CDC14C	CDC14 cell division cycle 14 homolog B (S. cerevisiae) /// CDC14 cell division cycle 14 homolog C (S. cerevisiae)
242	resistance	203713_s_at	5.28	LLGL2	lethal giant larvae homolog 2 (Drosophila)
