# Supplementary material for: The NRF2-mediated oxidative stress response pathway is associated with tumor cell resistance to arsenic trioxide across the NCI-60 panel
Source: BMC Med Genomics. 2010 Aug 13;3:37. doi: 10.1186/1755-8794-3-37 (PMC2939609; doi:10.1186/1755-8794-3-37)
Supplement: Additional file 3 — Gene products in network analysis. Lists all the 317 proteins contained within the large interactome. Each protein is listed as either its baseline expression level statistically associated with arsenic resistance/sensitivity, or it interacts with the directly associated transcripts. Corresponding gene symbols, gene names, gene IDs, and other relative information are included. Proteins within the three most significant sub-networks are also identified. [file 1755-8794-3-37-S3.PDF]

Additional File 3: gene products in network analysis

No.	Symbol	Entrez Gene Name	Gene ID	Association	Location	Family	Present in sub-network
1	ALDH3A1	aldehyde dehydrogenase 3 family, memberA1	205623_at	Resistance	Cytoplasm	enzyme	Yes
2	ALDH3A2	aldehyde dehydrogenase 3 family, member A2	210544_s_at	Resistance	Cytoplasm	enzyme	Yes
3	ALDH3B1	aldehyde dehydrogenase 3 family, member B1	205640_at	Resistance	Cytoplasm	enzyme	Yes
4	AMFR	autocrine motility factor receptor	202204_s_at	Resistance	Plasma Membrane	transmembrane receptor	Yes
5	ANXA2	annexin A2	213503_x_at	Resistance	Plasma Membrane	other	Yes
6	BCL2L14	BCL2-like 14 (apoptosis facilitator)	221241_s_at	Resistance	Cytoplasm	other	Yes
7	CAPNS1	calpain, small subunit 1	200001_at	Resistance	Cytoplasm	peptidase	Yes

Additional File 3: gene products in network analysis

8	CCNO	cyclin O	210021_s_at	Resistance	Nucleus	enzyme	Yes
9	CD9	CD9 molecule	201005_at	Resistance	Plasma Membrane	other	Yes
10	CTBP2	C-terminal binding protein 2	210835_s_at	Resistance	Nucleus	transcription regulator	Yes
11	CTNND1	catenin (cadherin-associated protein), delta 1	208862_s_at	Resistance	Nucleus	other	Yes
12	CYP2J2	cytochrome P450, family 2, subfamily J, polypeptide 2	205073_at	Resistance	Cytoplasm	enzyme	Yes
13	ELF3	E74-like factor 3 (ets domain transcription factor, epithelial-specific)	210827_s_at	Resistance	Nucleus	transcription regulator	Yes
14	F12	coagulation factor XII (Hageman factor)	205774_at	Resistance	Extracellular Space	peptidase	Yes
15	F2RL1	coagulation factor II (thrombin) receptor-like 1	206429_at	Resistance	Plasma Membrane	G-protein coupled receptor	Yes

Additional File 3: gene products in network analysis

16	FLNB	filamin B, beta	208614_s_at	Resistance	Cytoplasm	other	Yes
17	FTH1	ferritin, heavy polypeptide 1	200748_s_at	Resistance	Cytoplasm	enzyme	Yes
18	G6PD	glucose-6-phosphate dehydrogenase	202275_at	Resistance	Cytoplasm	enzyme	Yes
19	GCLC	glutamate-cysteine ligase, catalytic subunit	202922_at	Resistance	Cytoplasm	enzyme	Yes
20	GIPC1	GIPC PDZ domain containing family, member 1	207525_s_at	Resistance	Cytoplasm	other	Yes
21	GLI2	GLI family zinc finger 2	207034_s_at	Resistance	Nucleus	transcription regulator	Yes
22	GRB7	growth factor receptor-bound protein 7	210761_s_at	Resistance	Plasma Membrane	other	Yes
23	HTATIP2	HIV-1 Tat interactive protein 2, 30kDa	209448_at	Resistance	Nucleus	transcription regulator	Yes

Additional File 3: gene products in network analysis

24	ID1	inhibitor of DNA binding 1, dominant negative helix-loop-helix protein	208937_s_at	Resistance	Nucleus	transcription regulator	Yes
25	IRF6	interferon regulatory factor 6	202597_at	Resistance	Nucleus	transcription regulator	Yes
26	ITGA2	integrin, alpha 2 (CD49B, alpha 2 subunit of VLA-2 receptor)	205032_at	Resistance	Plasma Membrane	other	Yes
27	ITGB4	integrin, beta 4	204990_s_at	Resistance	Plasma Membrane	transmembrane receptor	Yes
28	KLF4	Kruppel-like factor 4 (gut)	221841_s_at	Resistance	Nucleus	transcription regulator	Yes
29	KLF5	Kruppel-like factor 5 (intestinal)	209212_s_at	Resistance	Nucleus	transcription regulator	Yes
30	KRT18	keratin 18	201596_x_at	Resistance	Cytoplasm	other	Yes
31	KRT19	keratin 19	201650_at	Resistance	Cytoplasm	other	Yes

Additional File 3: gene products in network analysis

32	KRT8	keratin 8	209008_x_at	Resistance	Cytoplasm	kinase	Yes
33	LAMC2	laminin, gamma 2	202267_at	Resistance	Extracellular Space	other	Yes
34	LPAR2	lysophosphatidic acid receptor 2	206723_s_at	Resistance	Plasma Membrane	G-protein coupled receptor	Yes
35	MST1R	macrophage stimulating 1 receptor (c-met-related tyrosine kinase)	205455_at	Resistance	Plasma Membrane	kinase	Yes
36	MSX2	msh homeobox 2	205555_s_at	Resistance	Nucleus	transcription regulator	Yes
37	NOL3	nucleolar protein 3 (apoptosis repressor with CARD domain)	221566_s_at	Resistance	Nucleus	other	Yes
38	NQO1	NAD(P)H dehydrogenase, quinone 1	201468_s_at	Resistance	Cytoplasm	enzyme	Yes
39	PLEKHA6	pleckstrin homology domain containing, family A member 6	205093_at	Resistance	unknown	other	Yes

Additional File 3: gene products in network analysis

40	PLXNB2	plexin B2	208890_s_at	Resistance	Plasma Membrane	other	Yes
41	PPAP2C	phosphatidic acid phosphatase type 2C	209529_at	Resistance	Plasma Membrane	phosphatase	Yes
42	PPARG	peroxisome proliferator-activated receptor gamma	208510_s_at	Resistance	Nucleus	ligand-dependent nuclear receptor	Yes
43	PTK6	PTK6 protein tyrosine kinase 6	206482_at	Resistance	Cytoplasm	kinase	Yes
44	S100A10	S100 calcium binding protein A10	200872_at	Resistance	Cytoplasm	other	Yes
45	SDC1	syndecan 1	201287_s_at	Resistance	Plasma Membrane	other	Yes
46	SERPINB5	serpin peptidase inhibitor, clade B (ovalbumin), member 5	204855_at	Resistance	Extracellular Space	other	Yes
47	SFN	stratifin	209260_at	Resistance	Cytoplasm	other	Yes

Additional File 3: gene products in network analysis

48	SLC6A2	solute carrier family 6 (neurotransmitter transporter, noradrenalin), member 2	215715_at	Resistance	Plasma Membrane	transporter	Yes
49	SMAD3	SMAD family member 3	205398_s_at	Resistance	Nucleus	transcription regulator	Yes
50	SQSTM1	sequestosome 1	201471_s_at	Resistance	Cytoplasm	transcription regulator	Yes
51	TACSTD1	epithelial cell adhesion molecule	201839_s_at	Resistance	Plasma Membrane	other	Yes
52	TGFA	transforming growth factor, alpha	205015_s_at	Resistance	Extracellular Space	growth factor	Yes
53	TPD52L1	tumor protein D52-like 1	203786_s_at	Resistance	Cytoplasm	other	Yes
54	TXN	thioredoxin	208864_s_at	Resistance	Cytoplasm	enzyme	Yes
55	TXNRD1	thioredoxin reductase 1	201266_at	Resistance	Cytoplasm	enzyme	Yes

Additional File 3: gene products in network analysis

56	VAV2	vav 2 guanine nucleotide exchange factor	205536_at	Resistance	Extracellular Space	cytokine	Yes
57	WISP2	WNT1 inducible signaling pathway protein 2	205792_at	Resistance	Extracellular Space	growth factor	Yes
58	ARID1A	AT rich interactive domain 1A (SWI-like)	210649_s_at	Sensitivity	Nucleus	transcription regulator	Yes
59	E2F3	E2F transcription factor 3	203693_s_at	Sensitivity	Nucleus	transcription regulator	Yes
60	HMGN2	high-mobility group nucleosomal binding domain 2	208668_x_at	Sensitivity	Nucleus	other	Yes
61	IFI16	interferon, gamma-inducible protein 16	208965_s_at	Sensitivity	Nucleus	transcription regulator	Yes
62	JARID2	jumonji, AT rich interactive domain 2	203298_s_at	Sensitivity	Nucleus	transcription regulator	Yes
63	MTF2	metal response element binding transcription factor 2	203346_s_at	Sensitivity	Nucleus	transcription regulator	Yes

Additional File 3: gene products in network analysis

64	NASP	nuclear autoantigenic sperm protein (histone-binding)	201969_at	Sensitivity	Nucleus	other	Yes
65	PCM1	pericentriolar material 1	214937_x_at	Sensitivity	Cytoplasm	other	Yes
66	PLEKHO1	pleckstrin homology domain containing, family O member 1	218223_s_at	Sensitivity	Plasma Membrane	other	Yes
67	SMARCC1	SWI/SNF related, matrix associated, actin dependent regulator of chromatin, subfamily c, member 1	201074_at	Sensitivity	Nucleus	transcription regulator	Yes
68	STMN1	stathmin 1	200783_s_at	Sensitivity	Cytoplasm	other	Yes
69	TOP2B	topoisomerase (DNA) II beta 180kDa	211987_at	Sensitivity	Nucleus	enzyme	Yes
70	ZEB1	zinc finger E-box binding homeobox 1	212758_s_at	Sensitivity	Nucleus	transcription regulator	Yes
71	Akt				unknown	group	Yes

Additional File 3: gene products in network analysis

72	ALDH				unknown	group	Yes
73	ALP				unknown	group	Yes
74	Ap1				unknown	complex	Yes
75	Calpain				unknown	complex	Yes
76	Cbp/p300				unknown	group	Yes
77	Ck2				unknown	complex	Yes
78	Cyclin A				unknown	group	Yes
79	Cyclin E				unknown	group	Yes

Additional File 3: gene products in network analysis

80	E2f				unknown	group	Yes
81	ERK				unknown	group	Yes
82	hCG				unknown	complex	Yes
83	Histone h3				unknown	group	Yes
84	IL1				unknown	group	Yes
85	IL12				unknown	complex	Yes
86	Insulin				unknown	group	Yes
87	Integrin				unknown	complex	Yes

Additional File 3: gene products in network analysis

88	Jnk				unknown	group	Yes
89	LDL				unknown	complex	Yes
90	Mapk				unknown	group	Yes
91	Nfat				unknown	group	Yes
92	NFkB				unknown	complex	Yes
93	P38 MAPK				unknown	group	Yes
94	Pdgf				unknown	complex	Yes
95	PDGF BB				unknown	complex	Yes

96	PI3K				unknown	complex	Yes
97	Pkc(s)				unknown	group	Yes
98	PP2A				unknown	complex	Yes
99	Proteasome				unknown	complex	Yes
100	Raf				unknown	group	Yes
101	Rb				unknown	group	Yes
102	RNA polymerase II				unknown	complex	Yes
103	Smad				unknown	complex	Yes

Additional File 3: gene products in network analysis

104	Ubiquitin				unknown	group	Yes
105	Vegf				unknown	group	Yes
106	ABCC1	ATP-binding cassette, sub-family C (CFTR/MRP), member 1	202804_at	Resistance	Plasma Membrane	transporter	
107	ABCC3	ATP-binding cassette, sub-family C (CFTR/MRP), member 3	209641_s_at	Resistance	Plasma Membrane	transporter	
108	ABLIM1	actin binding LIM protein 1	200965_s_at	Resistance	Cytoplasm	other	
109	AFTPH	aftiphilin	217939_s_at	Resistance	Cytoplasm	other	
110	AGR2	anterior gradient homolog 2 (Xenopus laevis)	209173_at	Resistance	Extracellular Space	other	
111	AKR1C1	aldo-keto reductase family 1, member C1 (dihydrodiol dehydrogenase 1; 20-alpha (3-alpha)-hydroxysteroid dehydrogenase)	216594_x_at	Resistance	Cytoplasm	enzyme	

Additional File 3: gene products in network analysis

112	AKR1C2	aldo-keto reductase family 1, member C2 (dihydrodiol dehydrogenase 2; bile acid binding protein; 3-alpha hydroxysteroid dehydrogenase, type III)	209699_x_at	Resistance	Cytoplasm	enzyme	
113	AKR1C3	aldo-keto reductase family 1, member C3 (3-alpha hydroxysteroid dehydrogenase, type II)	209160_at	Resistance	Cytoplasm	enzyme	
114	ANKRD2	ankyrin repeat domain 2 (stretch responsive muscle)	221232_s_at	Resistance	Nucleus	transcription regulator	
115	ANXA3	annexin A3	209369_at	Resistance	Cytoplasm	enzyme	
116	ASL	argininosuccinate lyase	204608_at	Resistance	Cytoplasm	enzyme	
117	ATP1B1	ATPase, Na+/K+ transporting, beta 1 polypeptide	201243_s_at	Resistance	Plasma Membrane	transporter	
118	CENTA1	ArfGAP with dual PH domains 1	90265_at	Resistance	Nucleus	other	
119	CHMP1A	chromatin modifying protein 1A	201933_at	Resistance	Extracellular Space	peptidase	

Additional File 3: gene products in network analysis

120	CLDN4	claudin 4	201428_at	Resistance	Plasma Membrane	transmembrane receptor	
121	CLDN7	claudin 7	202790_at	Resistance	Plasma Membrane	other	
122	CLN3	ceroid-lipofuscinosis, neuronal 3	209275_s_at	Resistance	Cytoplasm	other	
123	DCI	dodecenoyl-Coenzyme A delta isomerase (3,2 trans-enoyl-Coenzyme A isomerase)	209759_s_at	Resistance	Cytoplasm	enzyme	
124	DSC2	desmocollin 2	204751_x_at	Resistance	Plasma Membrane	other	
125	DSG2	desmoglein 2	217901_at	Resistance	Plasma Membrane	other	
126	EEF1A2	eukaryotic translation elongation factor 1 alpha 2	204540_at	Resistance	Cytoplasm	translation regulator	
127	EPB41L1	erythrocyte membrane protein band 4.1-like 1	212339_at	Resistance	Plasma Membrane	other	

Additional File 3: gene products in network analysis

128	ETHE1	ethylmalonic encephalopathy 1	204034_at	Resistance	Cytoplasm	other	
129	EVPL	envoplakin	204503_at	Resistance	Plasma Membrane	other	
130	FA2H	fatty acid 2-hydroxylase	219429_at	Resistance	unknown	enzyme	
131	FERMT1	fermitin family homolog 1 (Drosophila)	218796_at	Resistance	Plasma Membrane	other	
132	FLJ20489	solute carrier family 48 (heme transporter), member 1	48106_at	Resistance	unknown	other	
133	FUT3	fucosyltransferase 3 (galactoside 3(4)-L-fucosyltransferase, Lewis blood group)	214088_s_at	Resistance	Cytoplasm	enzyme	
134	GCNT3	glucosaminyl (N-acetyl) transferase 3, mucin type	219508_at	Resistance	Plasma Membrane	enzyme	
135	GDE1	glycerophosphodiester phosphodiesterase 1	202593_s_at	Resistance	Plasma Membrane	enzyme	

Additional File 3: gene products in network analysis

136	GJB3	gap junction protein, beta 3, 31kDa	215243_s_at	Resistance	Plasma Membrane	transporter	
137	GPRC5A	G protein-coupled receptor, family C, group 5, member A	203108_at	Resistance	Plasma Membrane	G-protein coupled receptor	
138	GSR	glutathione reductase	205770_at	Resistance	Cytoplasm	enzyme	
139	GULP1	GULP, engulfment adaptor PTB domain containing 1	215913_s_at	Resistance	Cytoplasm	other	
140	HFE	hemochromatosis	214647_s_at	Resistance	Plasma Membrane	transmembrane receptor	
141	INPP1	inositol polyphosphate-1-phosphatase	202794_at	Resistance	Cytoplasm	phosphatase	
142	KIAA0649	KIAA0649	203955_at	Resistance	unknown	other	
143	LAD1	ladinin 1	216641_s_at	Resistance	Extracellular Space	other	

Additional File 3: gene products in network analysis

144	LLGL2	lethal giant larvae homolog 2 (Drosophila)	203713_s_at	Resistance	Cytoplasm	other	
145	LOC26010	spermatogenesis associated, serine-rich 2-like	222154_s_at	Resistance	unknown	other	
146	LSR	lipolysis stimulated lipoprotein receptor	208190_s_at	Resistance	Nucleus	transcription regulator	
147	LTA4H	leukotriene A4 hydrolase	208771_s_at	Resistance	Cytoplasm	enzyme	
148	MALL	mal, T-cell differentiation protein-like	209373_at	Resistance	Plasma Membrane	other	
149	ME1	malic enzyme 1, NADP(+)-dependent, cytosolic	204058_at	Resistance	Cytoplasm	enzyme	
150	MGAT4B	mannosyl (alpha-1,3-)-glycoprotein beta-1,4-N-acetylglucosaminyltransferase, isozyme B	220189_s_at	Resistance	unknown	enzyme	
151	MRPL49	mitochondrial ribosomal protein L49	201717_at	Resistance	Cytoplasm	enzyme	

Additional File 3: gene products in network analysis

152	MTUS1	microtubule associated tumor suppressor 1	212093_s_at	Resistance	unknown	other	
153	MYO5C	myosin VC	218966_at	Resistance	unknown	other	
154	NUP37	nucleoporin 37kDa	218622_at	Resistance	Nucleus	transporter	
155	PCBD1	pterin-4 alpha-carbinolamine dehydratase/dimerization cofactor of hepatocyte nuclear factor 1 alpha	203557_s_at	Resistance	Nucleus	transcription regulator	
156	PDXK	pyridoxal (pyridoxine, vitamin B6) kinase	202671_s_at	Resistance	Cytoplasm	kinase	
157	PGD	phosphogluconate dehydrogenase	201118_at	Resistance	Cytoplasm	enzyme	
158	PKP3	plakophilin 3	209873_s_at	Resistance	Plasma Membrane	other	
159	PLEK2	pleckstrin 2	218644_at	Resistance	unknown	other	

Additional File 3: gene products in network analysis

160	PNPO	pyridoxamine 5'-phosphate oxidase	218511_s_at	Resistance	unknown	enzyme	
161	POLDIP2	polymerase (DNA-directed), delta interacting protein 2	217806_s_at	Resistance	Nucleus	other	
162	RAB25	RAB25, member RAS oncogene family	218186_at	Resistance	Cytoplasm	enzyme	
163	RHOD	ras homolog gene family, member D	31846_at	Resistance	Cytoplasm	enzyme	
164	SH2D3A	SH2 domain containing 3A	219513_s_at	Resistance	Cytoplasm	other	
165	SLC22A5	solute carrier family 22 (organic cation/carnitine transporter), member 5	205074_at	Resistance	Plasma Membrane	transporter	
166	SLC7A11	solute carrier family 7, (cationic amino acid transporter, y+ system) member 11	217678_at	Resistance	Plasma Membrane	transporter	
167	SPR	sepiapterin reductase (7,8-dihydrobiopterin:NADP+ oxidoreductase)	203458_at	Resistance	Cytoplasm	enzyme	

Additional File 3: gene products in network analysis

168	STK16	serine/threonine kinase 16	209622_at	Resistance	Cytoplasm	kinase	
169	TNK1	tyrosine kinase, non-receptor, 1	217149_x_at	Resistance	Cytoplasm	kinase	
170	TRIM15	tripartite motif-containing 15	210177_at	Resistance	unknown	other	
171	TSKU	tsukushin	218245_at	Resistance	unknown	other	
172	WWC1	WW and C2 domain containing 1	216074_x_at	Resistance	Cytoplasm	other	
173	ARID4B	AT rich interactive domain 4B (RBP1-like)	221230_s_at	Sensitivity	Nucleus	other	
174	BAT2D1	BAT2 domain containing 1	211946_s_at	Sensitivity	Cytoplasm	other	
175	DCP2	DCP2 decapping enzyme homolog (S. cerevisiae)	212919_at	Sensitivity	Nucleus	other	

Additional File 3: gene products in network analysis

176	FUBP1	far upstream element (FUSE) binding protein 1	212847_at	Sensitivity	Nucleus	transcription regulator	
177	HTATSF1	HIV-1 Tat specific factor 1	202602_s_at	Sensitivity	Nucleus	transcription regulator	
178	LZTFL1	leucine zipper transcription factor-like 1	218437_s_at	Sensitivity	unknown	other	
179	OGT	O-linked N-acetylglucosamine (GlcNAc) transferase (UDP-N-acetylglucosamine:polypeptide-N-acetylglucosaminyl transferase)	209240_at	Sensitivity	Cytoplasm	enzyme	
180	PRR3	proline rich 3	204795_at	Sensitivity	unknown	other	
181	PTPRC	protein tyrosine phosphatase, receptor type, C	212587_s_at	Sensitivity	Plasma Membrane	phosphatase	
182	SETMAR	SET domain and mariner transposase fusion gene	206554_x_at	Sensitivity	unknown	enzyme	
183	SFPQ	splicing factor proline/glutamine-rich (polypyrimidine tract binding protein associated)	214016_s_at	Sensitivity	Nucleus	other	

Additional File 3: gene products in network analysis

184	SH3BGRL	SH3 domain binding glutamic acid-rich protein like	201312_s_at	Sensitivity	unknown	other	
185	SNRPE	small nuclear ribonucleoprotein polypeptide E	203316_s_at	Sensitivity	Nucleus	other	
186	SON	SON DNA binding protein	214988_s_at	Sensitivity	Nucleus	other	
187	TRIM24	tripartite motif-containing 24	213301_x_at	Sensitivity	Nucleus	transcription regulator	
188	USP48	ubiquitin specific peptidase 48	220079_s_at	Sensitivity	Plasma Membrane	peptidase	
189	ZNF107	zinc finger protein 107	205739_x_at	Sensitivity	Nucleus	other	
190	12-hydroxyeicosatetraenoic acid				unknown	chemical - endogenous mammalian	
191	3alpha-hydroxysteroid dehydrogenase (A-specific)				unknown	group	

Additional File 3: gene products in network analysis

192	ADAM9	ADAM metallopeptidase domain 9 (meltrin gamma)			Plasma Membrane	peptidase	
193	AGT	angiotensinogen (serpin peptidase inhibitor, clade A, member 8)			Extracellular Space	other	
194	ALCAM	activated leukocyte cell adhesion molecule			Plasma Membrane	other	
195	ANXA7	annexin A7			Plasma Membrane	ion channel	
196	AP1B1	adaptor-related protein complex 1, beta 1 subunit			Cytoplasm	transporter	
197	AP1G2	adaptor-related protein complex 1, gamma 2 subunit			Cytoplasm	transporter	
198	AP3D1	adaptor-related protein complex 3, delta 1 subunit			Cytoplasm	transporter	
199	B2M	beta-2-microglobulin			Plasma Membrane	transmembrane receptor	

Additional File 3: gene products in network analysis

200	BAAT	bile acid Coenzyme A: amino acid N-acyltransferase (glycine N-choloyltransferase)			Cytoplasm	enzyme	
201	beta-estradiol				unknown	chemical - endogenous mammalian	
202	CAV2	caveolin 2			Plasma Membrane	other	
203	CCNG2	cyclin G2			Nucleus	other	
204	CD40LG	CD40 ligand			Extracellular Space	cytokine	
205	CGB	chorionic gonadotropin, beta polypeptide			Extracellular Space	other	
206	COL6A1	collagen, type VI, alpha 1			Extracellular Space	other	
207	CRAT	carnitine acetyltransferase			Cytoplasm	enzyme	

Additional File 3: gene products in network analysis

208	CTPS	CTP synthase			Nucleus	enzyme	
209	DBI	diazepam binding inhibitor (GABA receptor modulator, acyl-Coenzyme A binding protein)			Cytoplasm	other	
210	DGAT1	diacylglycerol O-acyltransferase homolog 1 (mouse)			Cytoplasm	enzyme	
211	DSC1	desmocollin 1			Plasma Membrane	other	
212	DSG1	desmoglein 1			Plasma Membrane	other	
213	DSP	desmoplakin			Plasma Membrane	other	
214	DYRK1B	dual-specificity tyrosine-(Y)-phosphorylation regulated kinase 1B			Nucleus	kinase	
215	EGF	epidermal growth factor (beta-urogastrone)			Extracellular Space	growth factor	

Additional File 3: gene products in network analysis

216	EGFR	epidermal growth factor receptor (erythroblastic leukemia viral (v-erb-b) oncogene homolog, avian)			Plasma Membrane	kinase	
217	EHHADH	enoyl-Coenzyme A, hydratase/3-hydroxyacyl Coenzyme A dehydrogenase			Cytoplasm	enzyme	
218	EXOSC5	exosome component 5			Nucleus	enzyme	
219	EXT2	exostoses (multiple) 2			Cytoplasm	enzyme	
220	F2RL2	coagulation factor II (thrombin) receptor-like 2			Plasma Membrane	G-protein coupled receptor	
221	FN1	fibronectin 1			Plasma Membrane	enzyme	
222	FOSL2	FOS-like antigen 2			Nucleus	transcription regulator	
223	FOXC2	forkhead box C2 (MFH-1, mesenchyme forkhead 1)			Nucleus	transcription regulator	

Additional File 3: gene products in network analysis

224	FSCN1	fascin homolog 1, actin-bundling protein (Strongylocentrotus purpuratus)			Cytoplasm	other	
225	FSH				unknown	complex	
226	FSHB	follicle stimulating hormone, beta polypeptide			Extracellular Space	other	
227	FST	follistatin			Extracellular Space	other	
228	GARS	glycyl-tRNA synthetase			Cytoplasm	enzyme	
229	GBE1	glucan (1,4-alpha-), branching enzyme 1			Cytoplasm	enzyme	
230	GFPT1	glutamine-fructose-6-phosphate transaminase 1			Cytoplasm	enzyme	
231	growth factor receptor				unknown	group	

232	GTF2E1	general transcription factor IIE, polypeptide 1, alpha 56kDa			Nucleus	transcription regulator	
233	GTF2F2	general transcription factor IIF, polypeptide 2, 30kDa			Nucleus	transcription regulator	
234	HMGN1	high-mobility group nucleosome binding domain 1			Nucleus	transcription regulator	
235	HNF1A	HNF1 homeobox A			Nucleus	transcription regulator	
236	HNF4A	hepatocyte nuclear factor 4, alpha			Nucleus	transcription regulator	
237	HOXA9	homeobox A9			Nucleus	transcription regulator	
238	HOXB2	homeobox B2			Nucleus	transcription regulator	
239	HOXC10	homeobox C10			Nucleus	transcription regulator	

Additional File 3: gene products in network analysis

240	HPSE	heparanase			Plasma Membrane	enzyme	
241	HSD17B1	hydroxysteroid (17-beta) dehydrogenase 1			Cytoplasm	enzyme	
242	HSD3B1	hydroxy-delta-5-steroid dehydrogenase, 3 beta- and steroid delta-isomerase 1			Cytoplasm	enzyme	
243	HSPA4	heat shock 70kDa protein 4			Cytoplasm	other	
244	IL4	interleukin 4			Extracellular Space	cytokine	
245	INS1	insulin I			Extracellular Space	other	
246	ITGB1	integrin, beta 1 (fibronectin receptor, beta polypeptide, antigen CD29 includes MDF2, MSK12)			Plasma Membrane	transmembrane receptor	
247	ITGB6	integrin, beta 6			Plasma Membrane	other	

Additional File 3: gene products in network analysis

248	ITGB7	integrin, beta 7			Plasma Membrane	transmembrane receptor	
249	ITGB8	integrin, beta 8			Plasma Membrane	other	
250	KIF1B	kinesin family member 1B			Cytoplasm	transporter	
251	KRT1	keratin 1			Cytoplasm	other	
252	LGALS3	lectin, galactoside-binding, soluble, 3			Extracellular Space	other	
253	LIG1	ligase I, DNA, ATP-dependent			Nucleus	enzyme	
254	LRSAM1	leucine rich repeat and sterile alpha motif containing 1			Cytoplasm	other	
255	LSM3	LSM3 homolog, U6 small nuclear RNA associated (S. cerevisiae)			Nucleus	other	

Additional File 3: gene products in network analysis

256	LSM4	LSM4 homolog, U6 small nuclear RNA associated (S. cerevisiae)			Nucleus	other	
257	LSM5	LSM5 homolog, U6 small nuclear RNA associated (S. cerevisiae)			Cytoplasm	other	
258	MGAT3	mannosyl (beta-1,4-)-glycoprotein beta-1,4-N-acetylglucosaminyltransferase			Cytoplasm	enzyme	
259	MGST1	microsomal glutathione S-transferase 1			Cytoplasm	enzyme	
260	MYO5A	myosin VA (heavy chain 12, myoxin)			Cytoplasm	enzyme	
261	MYO5B	myosin VB			Cytoplasm	enzyme	
262	NAB2	NGFI-A binding protein 2 (EGR1 binding protein 2)			Nucleus	transcription regulator	
263	NBR1	neighbor of BRCA1 gene 1			unknown	other	

Additional File 3: gene products in network analysis

264	NUP85	nucleoporin 85kDa			Cytoplasm	other	
265	PAH	phenylalanine hydroxylase			Cytoplasm	enzyme	
266	PARD6A	par-6 partitioning defective 6 homolog alpha (C. elegans)			Plasma Membrane	other	
267	PCNA	proliferating cell nuclear antigen			Nucleus	other	
268	PERP (includes EG:64065)	PERP, TP53 apoptosis effector			Plasma Membrane	other	
269	PHKB (includes EG:5257)	phosphorylase kinase, beta			Cytoplasm	kinase	
270	Pka				unknown	complex	
271	PLAGL1	pleiomorphic adenoma gene-like 1			Nucleus	transcription regulator	

Additional File 3: gene products in network analysis

272	POLD2	polymerase (DNA directed), delta 2, regulatory subunit 50kDa			Nucleus	enzyme	
273	POLD3	polymerase (DNA-directed), delta 3, accessory subunit			Nucleus	transcription regulator	
274	POLD4	polymerase (DNA-directed), delta 4			Nucleus	enzyme	
275	PPP1CA	protein phosphatase 1, catalytic subunit, alpha isoform			Cytoplasm	phosphatase	
276	PRG2 (includes EG:5553)	proteoglycan 2, bone marrow (natural killer cell activator, eosinophil granule major basic protein)			Extracellular Space	other	
277	PRKCZ	protein kinase C, zeta			Cytoplasm	kinase	
278	PSMC3	proteasome (prosome, macropain) 26S subunit, ATPase, 3			Nucleus	transcription regulator	
279	PTPRCAP	protein tyrosine phosphatase, receptor type, C-associated protein			Plasma Membrane	other	

Additional File 3: gene products in network analysis

280	RAB11FIP2	RAB11 family interacting protein 2 (class I)			Cytoplasm	other	
281	RAI2	retinoic acid induced 2			unknown	other	
282	Ras				unknown	group	
283	RDBP	RD RNA binding protein			Nucleus	other	
284	REST	RE1-silencing transcription factor			Nucleus	transcription regulator	
285	retinoic acid				unknown	chemical - endogenous mammalian	
286	RGS19	regulator of G-protein signaling 19			Cytoplasm	other	
287	S100A6	S100 calcium binding protein A6			Cytoplasm	transporter	

Additional File 3: gene products in network analysis

288	SF3B2	splicing factor 3b, subunit 2, 145kDa			Nucleus	other	
289	SFRS10	transformer 2 beta homolog (Drosophila)			Nucleus	other	
290	SLC3A2	solute carrier family 3 (activators of dibasic and neutral amino acid transport), member 2			Plasma Membrane	transporter	
291	SMAD7	SMAD family member 7			Nucleus	transcription regulator	
292	SNCG	synuclein, gamma (breast cancer-specific protein 1)			Cytoplasm	other	
293	SNRPA	small nuclear ribonucleoprotein polypeptide A			Nucleus	other	
294	SNRPD3	small nuclear ribonucleoprotein D3 polypeptide 18kDa			Nucleus	other	
295	SOX6	SRY (sex determining region Y)-box 6			Nucleus	transcription regulator	

Additional File 3: gene products in network analysis

296	SSTR1	somatostatin receptor 1			Plasma Membrane	G-protein coupled receptor	
297	STAMBP	STAM binding protein			Nucleus	enzyme	
298	SUPT5H	suppressor of Ty 5 homolog (S. cerevisiae)			Nucleus	transcription regulator	
299	T3-TR-RXR				unknown	complex	
300	TARS	threonyl-tRNA synthetase			Nucleus	enzyme	
301	testosterone				unknown	chemical - endogenous mammalian	
302	TGFB1	transforming growth factor, beta 1			Extracellular Space	growth factor	
303	TGFBR1	transforming growth factor, beta receptor 1			Plasma Membrane	kinase	

Additional File 3: gene products in network analysis

304	TGIF1	TGFB-induced factor homeobox 1			Nucleus	transcription regulator	
305	TNF	tumor necrosis factor (TNF superfamily, member 2)			Extracellular Space	cytokine	
306	TP53	tumor protein p53			Nucleus	transcription regulator	
307	TP53I3	tumor protein p53 inducible protein 3			unknown	enzyme	
308	TPSAB1	tryptase alpha/beta 1			Extracellular Space	peptidase	
309	TRAF2	TNF receptor-associated factor 2			Cytoplasm	other	
310	trans-(±)-3,5-cyclohexadiene-1,2-diol				unknown	chemical - endogenous mammalian	
311	Trans-1,2-dihydrobenzene-1,2-diol dehydrogenase				unknown	group	

Additional File 3: gene products in network analysis

312	UBQLN4	ubiquilin 4			Cytoplasm	other	
313	UCHL5	ubiquitin carboxyl-terminal hydrolase L5			Cytoplasm	peptidase	
314	UGT1A9 (includes EG:54600)	UDP glucuronosyltransferase 1 family, polypeptide A9			Cytoplasm	enzyme	
315	USP8	ubiquitin specific peptidase 8			Cytoplasm	peptidase	
316	WDR77	WD repeat domain 77			Nucleus	other	
317	YWHAZ	tyrosine 3-monooxygenase/tryptophan 5-monooxygenase activation protein, zeta polypeptide			Cytoplasm	enzyme	
