# Supplementary material for: The NRF2-mediated oxidative stress response pathway is associated with tumor cell resistance to arsenic trioxide across the NCI-60 panel
Source: BMC Med Genomics. 2010 Aug 13;3:37. doi: 10.1186/1755-8794-3-37 (PMC2939609; doi:10.1186/1755-8794-3-37)
Supplement: Additional file 4 — 64 common biological functions enriched in arsenic susceptibility associated networks. Lists all the 64 biological functions enriched in arsenic susceptibility associated networks. The functional category, p-value, and arsenic susceptibility-associated molecules within these functions are included. [file 1755-8794-3-37-S4.PDF]

Additional File 4: 64 common biological functions

Rank	Category	p-value	Molecules
1	Cell Death	2.62E-06	F2RL1, SFN, GULP1, S100A10, HTATIP2, KLF5, TXNRD1, TPD52L1, KRT18, VAV2, ITGA2, SERPINB5, PPARG, DSG2, MST1R, NQO1, OGT, NOL3, HFE, AGPAT2, FTH1, CYP2J2, G6PD, ELF3, FUBP1, SMARCC1, GSR, CLN3, CD9, KLF4, ID1, KRT19, STMN1, GCLC, ITGB4, BCL2L14, SQSTM1, PTPRC, SDC1, ABCC1, ALDH3A1, MSX2, TGFA, IFI16, CAPNS1, EEF1A2, ETHE1, ABCC3, TXN, E2F3, SMAD3, SNRPE, KRT8, CLDN4, CTBP2, SON, SETMAR
2	Cellular Movement	2.96E-06	STMN1, F2RL1, WISP2, ITGB4, S100A10, PTK6, SDC1, ABCC1, PLXNB2, MSX2, GRB7, TGFA, ITGA2, VAV2, RHOD, SERPINB5, CAPNS1, PPARG, MST1R, TXN, E2F3, SMAD3, GIPC1, LAMC2, CYP2J2, ANXA2, G6PD, ELF3, CD9, TOP2B, CTBP2, CLDN4, FLNB, KLF4, PKP3, ID1, LPAR2
3	Cell-To-Cell Signaling and Interaction	5.71E-06	ZEB1, STMN1, F12, ITGB4, WISP2, PTPRC, S100A10, SDC1, SLC6A2, FUT3, CTNND1, KRT18, TGFA, RHOD, ITGA2, SERPINB5, DSG2, PPARG, TXN, OGT, DSC2, SMAD3, LAMC2, ANXA2, CYP2J2, ELF3, ARID1A, CD9, KRT8, EPCAM
4	Tissue Development	5.71E-06	ZEB1, F12, ITGB4, WISP2, ATP1B1, PTPRC, SDC1, FUT3, CTNND1, TGFA, RHOD, ITGA2, SERPINB5, DSG2, PPARG, MST1R, TXN, DSC2, OGT, SMAD3, LAMC2, CYP2J2, ELF3, GLI2, ARID1A, CD9, KRT8, TOP2B, EPCAM

Additional File 4: 64 common biological functions

5	Cancer	1.55E-05	F2RL1, F12, WISP2, SFN, SLC7A11, ATP1B1, S100A10, PTK6, SLC6A2, HTATIP2, TXNRD1, KLF5, TPD52L1, PLXNB2, KRT18, HMGN2, ITGA2, SERPINB5, DSG2, PPARG, MST1R, NQO1, IRF6, AKR1C2, NOL3, CHMP1A, AGPAT2, CNNM4, FTH1, GIPC1, NUP37, CYP2J2, AKR1C3, G6PD, SMARCC1, FUBP1, ELF3, TMBIM1, CLN3, CD9, TOP2B, EPCAM, AMFR, KLF4, ID1, AKR1C1, ZEB1, KRT19, STMN1, TNK1, ITGB4, SQSTM1, PTPRC, AGR2, ABCC1, SDC1, SFPQ, BAT2D1, CTNND1, ALDH3A1, FA2H, DYRK1A, GRB7, TGFA, IFI16, RHOD, TRIM24, CAPNS1, ETHE1, ABCC3, TXN, E2F3, SMAD3, SNRPE, NASP, ASPH, LAMC2, ANXA2, GLI2, LIMD2, ARID1A, PCM1, PLEK2, CTBP2, CLDN4, GPRC5A, PKP3, LPAR2
6	Cellular Growth and Proliferation	1.71E-05	F2RL1, F12, WISP2, SFN, SLC7A11, PTK6, TXNRD1, KLF5, PLXNB2, VAV2, ITGA2, SERPINB5, PPARG, MST1R, IRF6, FTH1, GIPC1, AKR1C3, CYP2J2, G6PD, ELF3, PLEKHO1, CLN3, CD9, EPCAM, KLF4, ID1, ZEB1, STMN1, PPAP2C, TNK1, ITGB4, PTPRC, CTNND1, ALDH3A1, MSX2, GRB7, TGFA, IFI16, PDXK, CAPNS1, TXN, E2F3, SMAD3, NASP, MTUS1, ANXA2, GLI2, KRT8, CLDN4, CTBP2, JARID2, PKP3, LPAR2
7	Cellular Development	3.05E-05	ZEB1, STMN1, TNK1, ITGB4, SFN, SFPQ, CTNND1, DYRK1A, MSX2, TGFA, ITGA2, IFI16, PPARG, MST1R, TRIM15, E2F3, IRF6, SMAD3, EVPL, ANXA2, AKR1C3, ELF3, GLI2, PLEKHO1, TMBIM1, CD9, KRT8, KLF4, PKP3, ID1
8	Gastrointestinal Disease	3.39E-05	AKR1C1, KRT19, F12, SFN, ITGB4, SLC7A11, PTPRC, S100A10, SDC1, HTATIP2, KLF5, TPD52L1, CTNND1, KRT18, HMGN2, TGFA, ITGA2, DSG2, PPARG, NQO1, MST1R, ABCC3, TXN, AKR1C2, CHMP1A, CNNM4, ASPH, ANXA2, AKR1C3, NUP37, GLI2, CLN3, CD9, PLEK2, TOP2B, CLDN4, EPCAM, KLF4, LPAR2

Additional File 4: 64 common biological functions

9	Embryonic Development	3.89E-05	ABCC3, KRT19, TRIM15, OGT, SMAD3, ABCC1, ALDH3A2, SMARCC1, GLI2, DYRK1A, CD9, MSX2, KRT8, TOP2B, KRT18, EPCAM, TGFA, ITGA2, KLF4, SERPINB5, SETMAR, CAPNS1, PPARG, MST1R
10	Reproductive System Disease	5.20E-05	SFN, ITGB4, ABCC1, SDC1, CTNND1, GRB7, KRT18, TGFA, IFI16, ITGA2, RHOD, SERPINB5, PPARG, NQO1, ABCC3, E2F3, IRF6, SMAD3, SNRPE, AGPAT2, NASP, CYP2J2, ELF3, CLN3, TOP2B, CLDN4, EPCAM, ID1
11	Tumor Morphology	5.20E-05	E2F3, ITGB4, SMAD3, PTPRC, SDC1, HTATIP2, EPCAM, TGFA, ITGA2, SERPINB5, PPARG, MST1R, ID1
12	Amino Acid Metabolism	6.74E-05	ABCC3, CLN3, FTH1, ABCC1, ANXA2

Additional File 4: 64 common biological functions

13	Drug Metabolism	6.74E-05	ABCC3, STMN1, F2RL1, GCLC, SLC7A11, FTH1, ABCC1, AKR1C3, SLC6A2, G6PD, GSR, ALDH3A1, NQO1
14	Molecular Transport	6.74E-05	AKR1C1, ABCC3, F2RL1, TXN, GCLC, LSR, FTH1, ABCC1, AKR1C3, SLC6A2, PGD, G6PD, ALDH3A1, SPR, VAV2, PPARG, NQO1
15	Small Molecule Biochemistry	6.74E-05	AKR1C1, ABCC3, F2RL1, STMN1, AKR1C2, OGT, GCLC, SLC7A11, FTH1, ABCC1, ANXA2, AKR1C3, CYP2J2, SLC6A2, PGD, G6PD, LTA4H, GSR, ALDH3A1, FA2H, CLN3, VAV2, PPARG, NQO1
16	Vitamin and Mineral Metabolism	6.74E-05	ABCC3, FTH1, PPARG, ABCC1

17	Hair and Skin Development and Function	8.84E-05	IRF6, OGT, SFN, EVPL, CTNND1, ALDH3A1, MSX2, KRT8, KRT18, EPCAM, TGFA, KLF4, ID1
18	Cell Cycle	1.24E-04	ZEB1, PPAP2C, EPB41L1, ITGB4, SFN, PTPRC, TPD52L1, CTNND1, ALDH3A1, TGFA, IFI16, ITGA2, SERPINB5, PPARG, TXN, E2F3, SMAD3, NASP, CHMP1A, FUBP1, GLI2, PCM1, ARID1A, TOP2B, JARID2, KLF4, ID1
19	Energy Production	1.41E-04	AKR1C1, TXN, ALDH3A1, SPR, AKR1C3, NQO1, PGD
20	Cellular Assembly and Organization	1.60E-04	TXN, SFN, ITGB4, LAMC2, ANXA2, SMARCC1, CLN3, ARID1A, CD9, KRT8, KRT18, HMGN2, KLF4, PPARG

Additional File 4: 64 common biological functions

21	Cellular Function and Maintenance	1.60E-04	TXN, DSC2, ITGB4, SMAD3, EPCAM, KRT18, RHOD, DSG2, PPARG
22	Dermatological Diseases and Conditions	1.60E-04	ZEB1, TXN, F12, ITGB4, SFN, SMAD3, HFE, FERMT1, LAMC2, ANX2P2, SLC6A2, GLI2, TOP2B, KRT8, KRT18, GJB3, PPARG, ID1
23	Genetic Disorder	1.60E-04	IRF6, DSC2, ITGB4, MSX2, ASL, KRT8, HFE, FLNB, PPARG, DSG2, LAMC2
24	Hepatic System Development and Function	1.60E-04	LSR, SMAD3, KRT8, ASL, KRT18, JARID2, PPARG

25	Lipid Metabolism	1.60E-04	AKR1C1, ABCC3, LTA4H, FA2H, AKR1C2, CLN3, PPARG, ABCC1, CYP2J2, AKR1C3
26	Metabolic Disease	1.60E-04	ASL, KRT8, HFE, PPARG
27	Skeletal and Muscular System Development and Function	1.60E-04	TXN, DSC2, WISP2, SMAD3, GLI2, PLEKHO1, MSX2, CD9, TOP2B, FLNB, IFI16, DSG2, PPARG
28	Post-Translational Modification	3.06E-04	TXN, ALDH3A1, FTH1, ANXA2

Additional File 4: 64 common biological functions

29	Protein Degradation	3.06E-04	TXN, ALDH3A1, CLN3, PTPRC, FTH1, SDC1
30	Organ Development	4.04E-04	PCBD1, ALDH3A1, LSR, DSC2, SMAD3, ASL, JARID2, TGFA, KLF4, DSG2
31	Visual System Development and Function	4.04E-04	PCBD1, ALDH3A1, TGFA
32	Organ Morphology	4.75E-04	TXN, F2RL1, DYRK1A, DSC2, MSX2, KRT8, DSG2

Additional File 4: 64 common biological functions

33	Reproductive System Development and Function	4.75E-04	KRT19, ELF3, DSC2, KRT8, TGFA, ITGA2, SERPINB5, DSG2
34	Tissue Morphology	4.75E-04	KRT19, TXN, F2RL1, GLI2, MSX2, SMAD3, KRT8, TGFA, KLF4, ABCC1
35	Gene Expression	6.57E-04	ABCC3, ARID4B, TXN, E2F3, DCP2, SMAD3, CHMP1A, ABCC1, HTATIP2, SMARCC1, MSX2, ARID1A, TGFA, KLF4, IFI16, VAV2, TRIM24, PPARG, ID1
36	Carbohydrate Metabolism	9.43E-04	ABCC3, G6PD, OGT, ABCC1, PGD

Additional File 4: 64 common biological functions

37	Connective Tissue Development and Function	9.92E-04	PPAP2C, F2RL1, E2F3, WISP2, SMAD3, KLF5, GLI2, CD9, MSX2, KRT8, KRT18, IFI16, PPARG
38	Nervous System Development and Function	1.56E-03	GLI2, DYRK1A, CD9, TOP2B, ATP1B1
39	Cell Morphology	1.75E-03	EPB41L1, STMN1, ITGB4, SFN, PTPRC, CTNND1, MSX2, KRT18, TGFA, RHOD, ITGA2, SERPINB5, PPARG, MST1R, E2F3, IRF6, SMAD3, ANXA2, G6PD, GLI2, CD9, KRT8, KLF4, RHOF, ID1
40	Endocrine System Disorders	1.75E-03	TXN, HFE, EPCAM, TGFA, PPARG, ANXA2

Additional File 4: 64 common biological functions

41	Cellular Compromise	1.91E-03	ALDH3A2, STMN1, SMARCC1, TXN, SFN, ARID1A, MSX2, KRT18, HFE, PPARG, ABCC1, NQO1
42	Respiratory System Development and Function	1.94E-03	F2RL1, TXN, CD9, IFI16
43	Hematological System Development and Function	2.32E-03	SMAD3, S100A10, PPARG, ANXA2
44	Immune Response	2.32E-03	SMAD3, S100A10, ANXA2

Additional File 4: 64 common biological functions

45	Immune and Lymphatic System Development and Function	2.32E-03	S100A10, PPARG, ANXA2
46	Digestive System Development and Function	2.47E-03	F2RL1, LSR, SMAD3, ASL, JARID2, ASPH
47	Organismal Injury and Abnormalities	2.94E-03	F2RL1, FA2H, GLI2, SMAD3, KRT8, ASPH, PPARG
48	Hematological Disease	3.30E-03	KLF5, TXNRD1, STMN1, PTPRC, TGFA, FTH1, VAV2, ITGA2, KLF4, NQO1

Additional File 4: 64 common biological functions

49	Hepatic System Disease	3.91E-03	ABCC3, F12, GLI2, KRT8, KRT18, ABCC1, CYP2J2
50	Cardiovascular Disease	4.25E-03	TXN, DSC2, NOL3, SERPINB5, DSG2
51	Inflammatory Disease	5.42E-03	ABCC3, ZEB1, STMN1, F2RL1, TOP2B, PTPRC, ABCC1, PPARG, SLC6A2
52	Neurological Disease	5.42E-03	F2RL1, STMN1, PLXNB2, CLN3, TOP2B, SQSTM1, PTPRC, TGFA, SLC6A2

Additional File 4: 64 common biological functions

53	Cellular Response to Therapeutics	5.42E-03	ABCC3, ABCC1
54	Protein Trafficking	1.14E-02	LSR, PPARG
55	Connective Tissue Disorders	1.27E-02	STMN1, TXN, SMAD3, KRT8, KRT18, FTH1, IFI16, CAPNS1, ABCC1, ID1
56	Developmental Disorder	1.27E-02	IRF6, MSX2, KRT8, FLNB, TGFA, ASPH

Additional File 4: 64 common biological functions

57	Endocrine System Development and Function	1.27E-02	KRT8, AKR1C3
58	Immunological Disease	1.27E-02	PTPRC, VAV2, ITGA2
59	Nucleic Acid Metabolism	1.27E-02	G6PD, VAV2, NQO1
60	Ophthalmic Disease	1.27E-02	TXN, TGFA

Additional File 4: 64 common biological functions

61	Organismal Development	1.27E-02	PPARG
62	RNA Post-Transcriptional Modification	1.27E-02	DCP2
63	Respiratory Disease	1.27E-02	ABCC3, F2RL1, GLI2, CTBP2, TGFA, PPARG
64	Skeletal and Muscular Disorders	1.27E-02	TXN, FUBP1, SMAD3, NOL3
