# Supplementary material for: The NRF2-mediated oxidative stress response pathway is associated with tumor cell resistance to arsenic trioxide across the NCI-60 panel
Source: BMC Med Genomics. 2010 Aug 13;3:37. doi: 10.1186/1755-8794-3-37 (PMC2939609; doi:10.1186/1755-8794-3-37)
Supplement: Additional file 5 — Ten sub-networks within the large interactome. Lists the top ten sub-networks within the large interactome. Networks were built on the "Focus Molecules", whose baseline expression levels are statistically associated with tumor cell susceptibility to arsenic trioxide. Molecules in the networks are either focus molecules (e.g. gene biomarkers of arsenic susceptibility) or molecules interact with them. P-values for the ten sub-networks are detailed. [file 1755-8794-3-37-S5.PDF]

Additional File 5: ten sub-networks

No.	Molecules in Network	-Log (p-value)	Focus Molecules
1	Ap1, BCL2L14, CTBP2, CTNND1, ELF3, ERK, F2RL1, FLNB, GCLC, GIPC1, GLI2, HMGN2, IL12, ITGA2, Jnk, KLF4, KLF5, KRT8, KRT18, KRT19, MST1R, MTF2, Nfat, P38 MAPK, PCM1, Pkc(s), PLXNB2, Raf, SFN, Smad, SMAD3, TXN, TXNRD1, VAV2, ZEB1	49	26
2	ALDH, ALDH3A1, ALDH3A2, ALDH3B1, ALP, AMFR, ARID1A, Cbp/p300, Ck2, Cyclin A, Cyclin E, E2f, E2F3, GRB7, HTATIP2, ID1, IFI16, JARID2, LPAR2, MSX2, NASP, NFkB, NOL3, NQO1, PLEKHA6, PPAP2C, PPARG, Proteasome, Rb, RNA polymerase II, SMARCC1, SQSTM1, TACSTD1, TOP2B, Ubiquitin	42	23
3	Akt, ANXA2, Calpain, CAPNS1, CCNO, CD9, CYP2J2, F12, FTH1, G6PD, hCG, Histone h3, IL1, Insulin, Integrin, IRF6, ITGB4, LAMC2, LDL, Mapk, Pdgf, PDGF BB, PI3K, PLEKHO1, PP2A, PTK6, S100A10, SDC1, SERPINB5, SLC6A2, STMN1, TGFA, TPD52L1, Vegf, WISP2	37	21
4	AP3D1, beta-estradiol, CLDN4, CLN3, CTPS, DSP, EXT2, FOXC2, FUBP1, GARS, GPRC5A, GSR, GTF2E1, GTF2F2, HMGN1, HSPA4, HTATIP2, HTATSF1, KRT19, OGT, RDBP, RNA polymerase II, S100A6, SFRS10, SLC22A5, SNCG, SPR, STK16, SUPT5H, TPD52L1, TRIM24, TSKU, WISP2, WWC1, YWHAZ	28	17
5	CENTA1, CHMP1A, CLDN7, DCI, DSC1, DSC2, DSG1, DSG2, EGF, EHHADH, G6PD, GFPT1, GLI2, growth factor receptor, Histone h3, INS1, KRT8, LOC26010, MTUS1, MYO5A, MYO5B, MYO5C, Pka, PKP3, PLAGL1, RAB25, RAB11FIP2, SETMAR, SF3B2, SH2D3A, SMAD3, SOX6, STAMBP, TGIF1, USP8	28	17
6	ADAM9, ALCAM, ANXA7, ARID4B, ATP1B1, CAV2, CCNG2, CD40LG, COL6A1, EGFR, EVPL, FERMT1, FLJ20489, FLNB, FSCN1, GLI2, GULP1, HOXC10, ITGB1, ITGB6, ITGB7, ITGB8, KRT1, KRT8, LAMC2, LSR, MALL, MGAT3, SH2D3A, SH3BGRL, SLC3A2, SLC7A11, TGFB1, TPSAB1, WISP2	25	16

Additional File 5: ten sub-networks

7	12-hydroxyeicosatetraenoic acid, AGT, ANKRD2, ANXA3, ASL, ATP1B1, DCP2, EPB41L1, ETHE1, FN1, GJB3, HSD3B1, IL4, INPP1, ITGB8, LIG1, LTA4H, NAB2, NUP37, NUP85, PCNA, PERP (includes EG:64065), POLD2, POLD3, POLD4, POLDIP2, PSMC3, Ras, RHOD, SERPINB5, SON, TGFBR1, TNK1, TP53, TP53I3	25	16
8	ABCC3, ALDH3A1, BAAT, BAT2D1, CRAT, EXOSC5, GBE1, HNF4A, HOXA9, HOXB2, KIAA0649, KIF1B, LGALS3, LRSAM1, LSM3, LSM4, LSM5, MGAT4B, MGST1, MRPL49, PGD, PLEK2, PNPO, PPP1CA, PRR3, PTPRC, PTPRCAP, retinoic acid, SFPQ, SNRPA, SNRPD3, SNRPE, TRIM15, UGT1A9 (includes EG:54600), WDR77	21	14
9	ABCC1, AFTPH, ANXA3, AP1B1, AP1G2, ATP1B1, B2M, CGB, DBI, DGAT1, F2RL2, FOSL2, FSH, FSHB, FST, FUT3, GDE1, HFE, HPSE, HSD17B1, HSD3B1, LAD1, LLGL2, LZTFL1, MGST1, NQO1, PARD6A, PDXK, PRG2 (includes EG:5553), PRKCZ, RGS19, SMAD7, testosterone, TNF, ZNF107	19	13
10	3alpha-hydroxysteroid dehydrogenase (A-specific), ABLIM1, AGR2, AKR1C1, AKR1C2, AKR1C3, DYRK1B, EEF1A2, FA2H, GBE1, GCNT3, HNF1A, HNF4A, ME1, MTF2, NBR1, PAH, PCBD1, PHKB (includes EG:5257), RAI2, REST, SSTR1, T3-TR-RXR, TARS, TRAF2, trans-(±)-3,5-cyclohexadiene-1,2-diol, Trans-1,2-dihydrobenzene-1,2-diol dehydrogenase, UBQLN4, UCHL5, UGT1A9 (includes EG:54600), USP48	18	12
