# Supplementary material for: The NRF2-mediated oxidative stress response pathway is associated with tumor cell resistance to arsenic trioxide across the NCI-60 panel
Source: BMC Med Genomics. 2010 Aug 13;3:37. doi: 10.1186/1755-8794-3-37 (PMC2939609; doi:10.1186/1755-8794-3-37)
Supplement: Additional file 6 — LDH release in NRF2 knock-down tumor cells. Lactate dehydrogenase (LDH) release was measured in NRF2 knock-down tumor (A549) cells (NRF2-KD) or control cells (expressing GFP reporter) exposed to inorganic arsenic.* indicates p < 0.05. [file 1755-8794-3-37-S6.PDF]

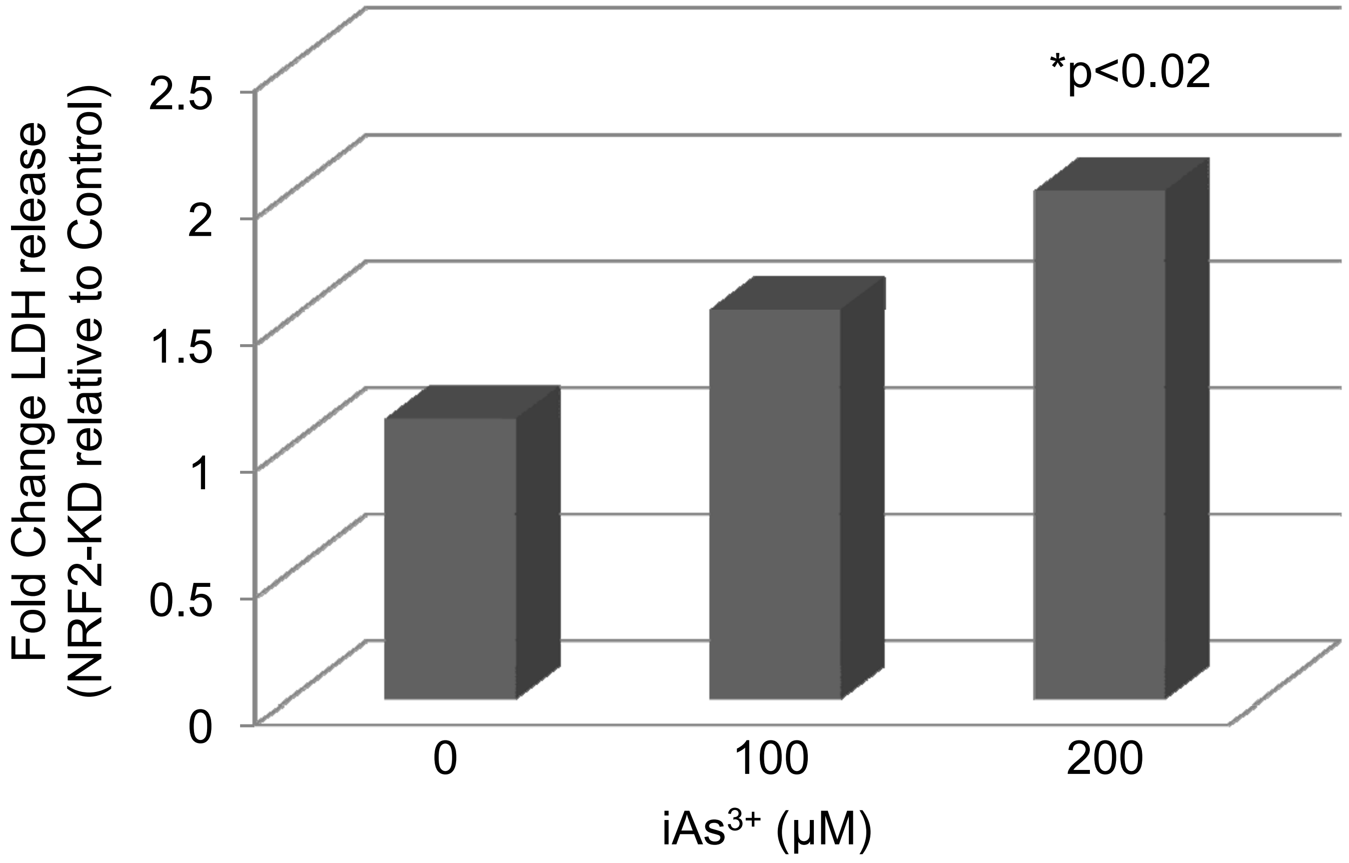

Additional File 6. Lactate dehydrogenase (LDH) release was measured in NRF2 knock-down tumor (A549) cells (NRF2-KD) or control cells exposed to inorganic arsenic. * indicates $p<0.05$.
